# Applications of Social Media and Digital Technologies in COVID-19 Vaccination: Scoping Review

**DOI:** 10.2196/40057

**Published:** 2023-02-10

**Authors:** Shujie Zang, Xu Zhang, Yuting Xing, Jiaxian Chen, Leesa Lin, Zhiyuan Hou

**Affiliations:** 1 School of Public Health Fudan University Shanghai China; 2 Global Health Institute Fudan University Shanghai China; 3 Department of Infectious Disease Epidemiology London School of Hygiene & Tropical Medicine London United Kingdom; 4 Laboratory of Data Discovery for Health (D24H) Hong Kong Science Park Hong Kong, SAR China

**Keywords:** social media, digital health, COVID-19, vaccination, review

## Abstract

**Background:**

Social media and digital technologies have played essential roles in disseminating information and promoting vaccination during the COVID-19 pandemic. There is a need to summarize the applications and analytical techniques of social media and digital technologies in monitoring vaccine attitudes and administering COVID-19 vaccines.

**Objective:**

We aimed to synthesize the global evidence on the applications of social media and digital technologies in COVID-19 vaccination and to explore their avenues to promote COVID-19 vaccination.

**Methods:**

We searched 6 databases (PubMed, Scopus, Web of Science, Embase, EBSCO, and IEEE Xplore) for English-language articles from December 2019 to August 2022. The search terms covered keywords relating to social media, digital technology, and COVID-19 vaccines. Articles were included if they provided original descriptions of applications of social media or digital health technologies/solutions in COVID-19 vaccination. Conference abstracts, editorials, letters, commentaries, correspondence articles, study protocols, and reviews were excluded. A modified version of the Appraisal Tool for Cross-Sectional Studies (AXIS tool) was used to evaluate the quality of social media–related studies. The review was undertaken with the guidance of the Preferred Reporting Items for Systematic Reviews and Meta-Analyses extension for Scoping Reviews.

**Results:**

A total of 178 articles were included in our review, including 114 social media articles and 64 digital technology articles. Social media has been applied for sentiment/emotion analysis, topic analysis, behavioral analysis, dissemination and engagement analysis, and information quality analysis around COVID-19 vaccination. Of these, sentiment analysis and topic analysis were the most common, with social media data being primarily analyzed by lexicon-based and machine learning techniques. The accuracy and reliability of information on social media can seriously affect public attitudes toward COVID-19 vaccines, and misinformation often leads to vaccine hesitancy. Digital technologies have been applied to determine the COVID-19 vaccination strategy, predict the vaccination process, optimize vaccine distribution and delivery, provide safe and transparent vaccination certificates, and perform postvaccination surveillance. The applied digital technologies included algorithms, blockchain, mobile health, the Internet of Things, and other technologies, although with some barriers to their popularization.

**Conclusions:**

The applications of social media and digital technologies in addressing COVID-19 vaccination–related issues represent an irreversible trend. Attention should be paid to the ethical issues and health inequities arising from the digital divide while applying and promoting these technologies.

## Introduction

The COVID-19 pandemic has greatly accelerated the applications of social media and digital technologies. As a digital tool that allows real-time information sharing, social media has become a platform to not only voice public opinions, perceptions, and attitudes toward public health policies or events but also help governments and the public to exchange information in a timely manner [1]. However, it can lead to the spread of misinformation and disinformation [2], which may adversely affect public responses to the pandemic. At the same time, the application of digital technologies has facilitated the management and responses of the COVID-19 pandemic and other emerging infectious diseases in ways that are difficult to achieve manually [3,4].

The use of social media “big data” for public health surveillance and behavior monitoring is a rapidly growing field, allowing researchers to understand public attitudes and behaviors toward vaccines and other health issues [1,5,6]. Many previous studies have used data from social media platforms, such as Twitter and Facebook, to analyze COVID-19 vaccine acceptance and responses to the COVID-19 infodemic, vaccination-related misinformation, and rumors [1,7-10]. Several studies have also explored applications of digital technologies for controlling the COVID-19 pandemic, such as COVID-19 planning and tracking, screening for infection, contact tracing, and clinical management through artificial intelligence algorithms, blockchain, the Internet of Things (IoT), and big data analytics [3,4,11]. Many countries have launched mobile health (mHealth) apps to support COVID-19 vaccination services, such as vaccination certification and health monitoring [12,13].

Widespread distribution, acceptance, and uptake of COVID-19 vaccines are crucial for reducing severity and deaths due to infections. During the first year of global vaccination efforts, COVID-19 vaccines are estimated to have saved 19.8 million lives [14]. However, COVID-19 vaccination faces various challenges, including the formation of national or state-level vaccination strategies; community-wide vaccine storage, distribution, and delivery; and changes in public acceptance and confidence in vaccines. Social media and digital technologies have great potentials for applications in addressing these vaccination challenges, yet there is a lack of a literature review summarizing these applications. Our scoping review aimed to synthesize the global evidence on the applications of social media and digital technologies in COVID-19 vaccination. We documented the forms of digital tools, analysis techniques, application fields, and findings for COVID-19 vaccination that would benefit the advancement of COVID-19 vaccination and other future vaccination campaigns.

## Methods

### Data Search and Screening

The scoping review was conducted following the Preferred Reporting Items for Systematic Reviews and Meta-Analyses extension for Scoping Reviews (PRISMA-ScR) [15]. The PRISMA-ScR checklist is shown in Multimedia Appendix 1. We searched 6 peer-reviewed databases (PubMed, Scopus, Web of Science, Embase, EBSCO, and IEEE Xplore) for English articles published from December 1, 2019, to August 17, 2022. The search terms covered keywords relating to social media, digital technology, and COVID-19 vaccine, as shown in Table 1. The detailed search strategy for each database is shown in Multimedia Appendix 2.

**Table 1:** Literature search terms for the review.

Category	Key search terms
Social media	“social media,” “mass media,” “social networking,” “social network,” “internet,” “webcast,” “blogging,” “online community,” “online post,” “facebook,” “twitter,” “youtube,” “Instagram,” “weibo,” “wechat,” “tiktok,” “line,” “reddit,” “whatsapp,” “telegram,” “mobile application*,” “mobile app,” “chat bots”
Digital technology	“digital,” “digital health,” “digital technology,” “digital platform*,” “big data,” “data sharing,” “cloud computing,” “block chain,” “artificial intelligence,” “AI,” “natural language,” “deep learning,” “machine learning,” “neural network,” “information technology,” “internet of thing*,” “IoT,” “crowdsourcing,” “telemedicine,” “telehealth,” “mhealth,” “mobile health,” “ehealth,” “telecommunication*,” “remote consultation,” “teleconsultation*,” “telesupport,” “telemonitoring”
COVID-19	“COVID-19,” “covid,” “coronavirus,” “coronavirus disease 2019,” “2019-nCov,” “Severe acute respiratory syndrome coronavirus 2,” “sars-cov-2”
Vaccine	“vaccin*,” “immunis*,” “immuniz*”
COVID-19 vaccine	“COVID-19 vaccin*”

Two reviewers independently conducted initial screening for titles/abstracts and final screening for full texts to decide whether an article met the inclusion criteria. When discrepancies for included articles emerged, they carried out discussions together to reach a consensus. Articles were included if they provided original descriptions of the applications of social media or digital health technologies/solutions in COVID-19 vaccination. Specifically, original studies with original data or results referring to social media or digital tools/interventions for COVID-19 vaccination were included, and included studies covered experimental studies, cohort studies, case-control studies, observational studies (cross-sectional studies or surveys), case series/case studies, and description studies. The included studies involved at least one application of social media or digital tools/interventions in COVID-19 vaccination. We excluded studies that (1) investigated a non–COVID-19 vaccine, prevaccine development, or the COVID-19 pandemic instead of the COVID-19 vaccine; (2) did not investigate online media; and (3) did not focus on social media or digital technologies in COVID-19 vaccination. We also excluded the following study types: conference abstracts, editorials, letters, commentaries, correspondence articles, study protocols, and reviews.

### Quality Assessment

Due to the significant variation in the quality of social media–related studies, we used a modified version of the Appraisal Tool for Cross-Sectional Studies (AXIS tool in Multimedia Appendix 3) to evaluate their quality [16]. Two reviewers conducted this quality assessment, and the risk of bias was presented as “low risk,” “some concerns,” or “high risk.” Articles with a score of 12 or above were identified as having a low risk of bias and kept for our review. Since there was no assessment tool applicable for digital technology–related studies and no significant differences were found in their quality, we did not assess the quality of the included digital technology articles.

### Data Extraction and Analysis

For each included article, 2 researchers extracted data independently and discussed any discrepancies to reach a consensus. The extracted data included article information (title, first author, and journal), study period, study design, data sources, study population and sample size, information on social media (social media platforms, application domains, analysis technologies, and findings), information on digital technologies (names/types of digital technologies, application domains, and applications in detail), and future research/suggestions. The innovation features and generalizability of identified digital technologies/solutions were also evaluated and summarized. According to the report on digital technologies in health services from the Expert Panel on Effective Ways of Investing in Health (EXPH) [17], the innovation features of digital technologies/solutions could be supportive, complementing, innovative, or substitutive to existing/previous technologies. The generalizability covered the following 3 groups: not possible (strict bond to the context in which it was developed), local (scalability is limited to a local regional context), and global (no barriers to scalability for global adoption) [18].

This review was divided into the following 2 modules: applications of social media and applications of digital technologies/solutions in COVID-19 vaccination. After reviewing social media or digital technologies used in each article, we grouped their applications into several fields relating to COVID-19 vaccination and summarized these techniques and critical findings.

### Patient and Public Involvement

Patients or the public were not involved in the design, conduct, reporting, or dissemination plans of our research.

### Ethics Approval

Ethics approval was waived since this is a secondary analysis of published articles.

## Results

### Included Articles

**Figure 1:**
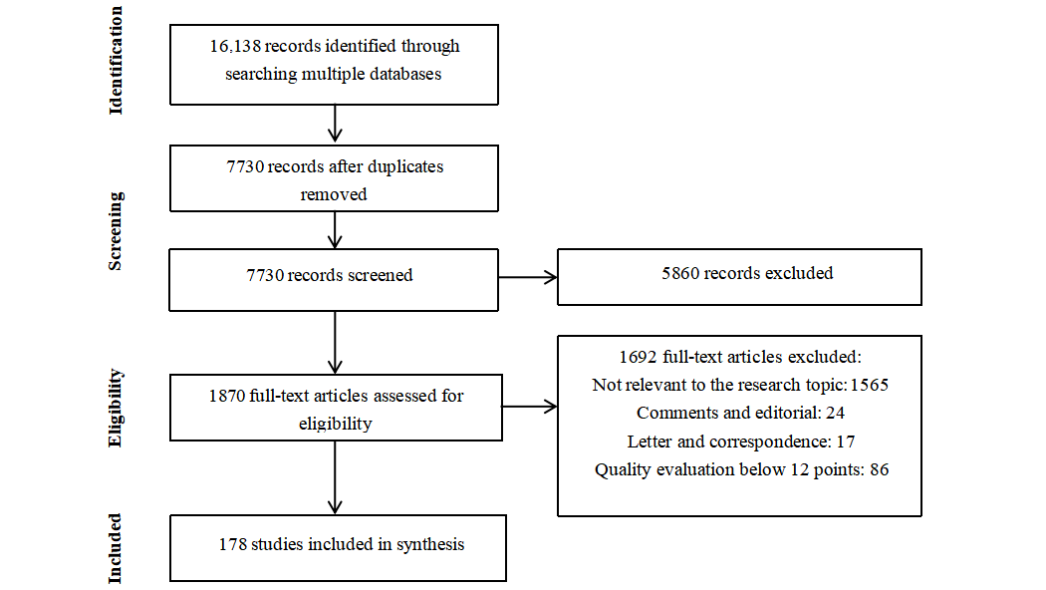
PRISMA (Preferred Reporting Items for Systematic Reviews and Meta-Analyses) flowchart.

After the eligibility assessment, 178 articles were included in this review, including 114 studies on social media applications with a low risk of bias and 64 on digital technologies for COVID-19 vaccination (Figure 1).

### Applications of Social Media in COVID-19 Vaccination

Social media applications in COVID-19 vaccination mainly involved Twitter (87/114), Facebook (10/114), and YouTube (9/114). The applications covered the following 5 aspects: sentiment/emotion analysis (70/114), topic analysis (53/114), behavioral analysis (3/114), dissemination and engagement analysis (9/114), and information quality analysis (7/114). Details of the included social media studies are presented in Multimedia Appendix 4.

#### Sentiment/Emotion Analysis

Seventy articles conducted sentiment/emotion analysis through social media applications to identify public sentiments and opinions toward COVID-19 vaccines. The approaches of sentiment analysis included lexicon-based approaches (n=40), machine learning approaches (n=19), hybrid methods (n=7), and manual coding classification (n=4). Forty studies applied the lexicon-based sentiment analysis method, using predefined lexicons annotated with sentiment polarities (eg, positive, negative, or neutral) to determine sentiments expressed in the parsed text [19-58]. TextBlob and VADER (Valence Aware Dictionary and Sentiment Reasoner) were 2 well-known rule-based lexical sentiment analyzers [19,25,26,33,37-41,43,44,46,49,51,53,56-58]. Moreover, 11 studies further predicted the emotion types expressed in the tweets [20,21,23,26,32,37,45,50,53,54,58], and 8 classified emotions as “trust, surprise, sadness, joy, anticipation, disgust, fear, and anger” using the National Research Council Sentiment Lexicon [20,23,32,37,45,50,54,58].

Nineteen sentiment analysis studies were based on machine learning, and they trained machine learning classifiers by annotating tweets in the data set [59-77]. Machine learning approaches used supervised classification algorithms to extract information regarding sentiment polarity. Classical machine learning models mainly included naïve Bayes, support vector machine, random forest, decision tree, and logistic regression [60,61,67,70,76]. Additional machine learning techniques for polarity classification were Microsoft Azure cognitive services, Amazon Web Services (AWS), and Baidu’s AipNLP [59,64,75]. Deep learning techniques mainly used convolutional neural networks, recurrent neural networks, bidirectional long short-term memory (LSTM), and Bidirectional Encoder Representations from Transformers [63,65,66,68,71,77]. 

Seven studies employed hybrid methods combining lexicons and machine learning for polarity classification [7,78-83]. Accuracy, precision, recall, and F1 score were typically used to evaluate the performance of classification models [61,68-70]. In terms of model performance, 3 studies showed that LSTM outperformed other classifiers [68,70,78].

These sentiment analysis studies showed that public sentiments were associated with real-time news, internet information, public health events, the number of COVID-19 cases, vaccine development, the pandemic, and announcements of political leaders or authorities [7,21,27,31,51,52,55,56,58,65]. Although public sentiments on COVID-19 vaccines varied significantly over time and geography [7,33,39], positive sentiments were more prevalent than negative ones regarding COVID-19 vaccines [7,19,20,22,25,29,33,41-44,46,47,51,57,60,62,65,70, 72,76,78,84,85], with trust and anticipation being the predominant emotions [20,23,32,37,50,54,58]. However, some other studies found that negative sentiments overwhelmed positive ones, with fear being the dominant emotion [53,59,64,66,71,73,79-82,86]. Positive sentiments were found to be mainly related to increased vaccine coverage, vaccine development, vaccination research, and health services [31,57,67,69], whereas negative sentiments were positively associated with increased COVID-19 cases, misinformation, conspiracy theories, and fear regarding vaccine safety [21,55]. Socioeconomically disadvantaged groups were more likely to hold polarized opinions on COVID-19 vaccines [22]. People with bad experiences during the pandemic were more likely to hold antivaccine opinions [22], whereas comments on posts from health media and hospitals had more positive attitudes [74]. Social media posts on Pfizer and Moderna vaccines appeared to be more positive than posts on COVID-19 vaccines from other manufacturers [24,77,83].

#### Topic Analysis

Fifty-three studies applied social media for topic analysis on COVID-19 vaccination. Topic analysis methods included latent Dirichlet allocation (LDA) topic modeling (n=24) [20,23,25,31,34,35,40,47,50,51,54-56,58,60,61,64,87-93], manual coding (n=17) [57,80,94-108], and other algorithms (n=12) [26,43,62,109-117]. Table 2 summarizes the provaccine and antivaccine topics on COVID-19 vaccines present on social media.

Topic analysis identified various attitudes and opinions toward COVID-19 and its vaccine, with main topics focusing on vaccination policy, vaccine development, vaccine administration and access, vaccination propaganda, vaccine efficacy and side effects, vaccine hesitancy, and conspiracy theories [26,43,47,50,55,61,64,90,100,101]. Vaccine objection and hesitancy were generally more prevalent than vaccine support [57], although opinion patterns differed by studies. Vaccine hesitancy mainly stemmed from safety concerns, mistrust in the government and pharmaceutical companies, lack of knowledge, conspiracy theories, skepticism about vaccine development and approval, vaccine ineffectiveness, and loss of freedom [20,25,43,55,56,87-89,93,96-98,100,106,111,112]. The dominant concerns regarding COVID-19 vaccines were safety issues and side effects, such as fear of death and allergic reactions to COVID-19 vaccines [56]. Pain, fever, and fatigue were the 3 most common adverse reactions reported by the public [109,117]. Antivaccine topics varied across social media platforms. For example, the activities of antivaxxers on Facebook and Twitter focused on distrust in the government and allegations regarding vaccination safety and effectiveness, while discussions on TikTok focused on individual freedom [105]. Topics on social media also changed over time. Liu et al found that the prevalence of tweets with positive behavioral intentions increased over time [93].

Additionally, public discussions were mainly driven by COVID-19 vaccine–related news, major social events, pandemic severity, and statements issued by authorities [23,26,34,51,58,87,89]. Regarding information sources, authoritative and reliable information disseminators, such as government agencies, major media outlets, and key opinion leaders, played massively influential roles in polarizing opinions, which can amplify or contain the spread of misinformation among target audiences. Positive discourses were more likely to interact with verified sources, such as news organizations, health professionals, and media/journalists, while negative discourses tended to interact with politicians and personal accounts [40,114].

**Table 2:** Provaccine and antivaccine topics from thematic analysis on social media articles.

Category and subcategory			Explanation
**Provaccine**			
	Safety and efficacy	Content on confidence in the safety or efficacy of vaccines.	
	Positive attitude	Content reflecting positive attitudes toward vaccines and government measures. It includes viewing vaccination as an act of dismantling systemic racism, being positive about the development of vaccines and antivirals, and expressing concerns on the antivaccine movement.	
	Criticizing antivaccine beliefs	Content that expresses support for vaccines by blaming antivaxxers. It includes accusing, ridiculing, and insulting antivaxxers for spreading falsehoods and misinformation.	
	Promotion	Content that advocates infection control measures and touts the prior success of immunizations.	
**Antivaccine**			
	Safety or effectiveness concerns	Content that lacks confidence in the safety or efficacy of vaccines, such as fear of health hazards, side effects, allergic reactions, or death attributable to vaccines; skepticism about vaccine trials; and inability of vaccines to prevent COVID-19.	
	Vaccine alternatives	Content that believes there are better immunization options than vaccines. It includes belief in God’s protection; protective behaviors, such as sunbathing, healthy eating, and exercise; and natural immunity from COVID-19 infection.	
	Morality and ethics	Content of vaccines offending liberty. It includes mandatory government policies, and loss of personal choice and freedom.	
	Misinformation	Content that disseminates false information about side effects, and vaccine production and transport. It includes scientific misinformation directly contrary to vaccine research and political misinformation of untrue government interventions.	
	Conspiracy	Content on scientific, political, or racial conspiracy theories. It includes deliberately created viruses, nonexistence of vaccines, microchips in vaccines, conflicts of interest between the government and pharmaceutical companies, genocide conspiracies, and right-wing politics.	
	Vaccine hesitancy	Content refers to delay in acceptance or refusal of vaccination despite availability of vaccination services.	

#### Behavioral Analysis

Three studies used social media to analyze behavioral intention and search behavior for COVID-19 vaccination [118-120]. Positive behavioral intention was influenced by reduced risk of infection, socioeconomic recovery, and normal life recovery. In contrast, negative behavioral intention was associated with misconceptions about vaccines and diseases, trust in natural immunity, distrust in the government and vaccines, and lack of knowledge [120]. Search interests regarding misinformation, generic information about vaccines, and availability of vaccines changed throughout the pandemic [118].

#### Dissemination and Engagement Analysis

Nine studies conducted dissemination and engagement analysis on social media regarding COVID-19 vaccination [19,51,106,114,121-125]. Social media engagement metrics included popularity based on the number of likes, commitment based on the number of comments, and virality based on the number of post shares. The betweenness centrality score is a traditional social network analysis technique used to discover the most influential users on social media [125]. Observing and analyzing the most attention-capturing tweets may help craft a better vaccine information policy. Compared to other social media platforms, Facebook was the most popular analyzed social media platform, followed by Twitter [106]. COVID-19 posts were more widely disseminated and showed more significant influence than non–COVID-19 posts [124]. Specifically, “antivaccine” groups were highly engaged in the COVID-19 vaccine discussion [121,122], and antivaccine sentiment was especially salient in the political right cluster [122]. Health care professionals had an essential role in supporting vaccine activities, and the highest active professional groups were pharmacists, nurses, physicians, and psychologists [125]. 

#### Information Quality Analysis

Seven studies assessed the reliability and quality of information about COVID-19 vaccination on social media according to the checklist and quality criteria for health information [125-131]. Reliability and credibility were assessed by the modified Health on the Net Foundation Code of Conduct (HONCode), and quality and reliability were evaluated according to DISCERN criteria. Studies showed that most videos were of high quality with good integrity, comprehensibility, relevance, depth, and accuracy of the information provided, and videos with factual information were higher in quality than those with nonfactual information [127,128,131]. Sources of high-quality videos were pharmaceutical companies, pharmacists, society organizations, and academics, while news provided a high percentage of low-quality videos [128]. COVID-19 vaccination FAQ websites provided quality information, but more effort should be taken to make the content more readable and to update the content [130]. The reputation, expertise, and presentation qualities of the authors were the main criteria for evaluating their credibility, while the credibility of antivaxxers as creators of vaccine-related information was very low [126].

### Applications of Digital Technologies in COVID-19 Vaccination

Among the included 64 articles (Multimedia Appendix 5), there were 5 types of digital technologies: algorithms (33/64), blockchain (21/64), mHealth (10/64), IoT (4/64), and other technologies, including online tools, biometrics, cloud storage, and digital twin (5/64). Traditional mathematical models, machine learning, and deep learning algorithms were classified as algorithm technologies. Mobile apps and mobile tracking through mobile and wireless technologies were classified as mHealth [132]. Blockchain was regarded as an essential technology. Most digital technology studies did not mention the research design but described a digital technology/solution for a specific field in COVID-19 vaccination. The applications covered the following 6 fields of COVID-19 vaccination (Table 3): strategy of COVID-19 vaccination (9/64), distribution and delivery of COVID-19 vaccines (22/64), model prediction of COVID-19 vaccination (6/64), COVID-19 vaccination services (11/64), certification of COVID-19 vaccination (13/64), and postvaccination surveillance (3/64).

**Table 3:** Applications of digital technologies in COVID-19 vaccination.

Field of COVID-19 vaccination	Algorithm	Blockchain	Mobile health	Internet of Things (IoT)	Others
Strategy of COVID-19 vaccination	XGBoost model [133,134] Genetic algorithms [135,136] Fuzzy logic system [137] Random forest [134] Logistic regression [134] Long short-term memory [138] Autoregressive Integrated Moving Average Model [138] Reinforcement learning [139] Disease propagation graph [140] Spatial artificial intelligence [141]	N/A^a^	N/A	N/A	N/A
Distribution and delivery of COVID-19 vaccines	No specifics [142,143] Spatiogeographical model, minimum-cost flow problem, and self-designed scheduling algorithm [144] Double Deep Q-Network, Advantage Actor Critic, Trust Region Policy Optimization, Actor-Critic using Kronecker-Factored Trust Region, and Linear Upper Confidence Bounds model [145] Long short-term memory [146,147] Gray Wolf Optimization [148] Variable neighborhood search [148,149] Whale Optimization Algorithm [149] Artificial neural network and convolutional neural network [150] Heuristic algorithm [151] Machine learning [152] Unsupervised self-organizing map, recurrent neural network, and Stochastic Mixture Density Network [147]	No specifics [142,152-154] Ethereum [155,156] Smart contract [156-161]	N/A	No specifics [142,143,162] Software-Defined Networking-IoT model [163]	5G-UAVCN [157] 6G-eRLLC [161]
Model prediction of COVID-19 vaccination	Logistic regression [164] Linear regression [165,166] AdaBoost [164,167] Boost classification [166] Decision tree [164,165] Random forest [164,166,167] Long short-term memory [168,169] DeepAR [169] Support vector machine [165] Autoregressive model [166] Extra trees [167] Gradient boosting [167] XGBoost [167]	N/A	N/A	N/A	N/A
COVID-19 vaccination services	AnyLogic model and a trained neural network [170] Accelerated Dual Ascent [171] Decreasing order-based algorithm, iterative random algorithm, and clustering algorithm [172] K-means clustering [173]	N/A	App [174-177] Personalized message/email [178]	N/A	Online tool [179] Cloud storage [180] Digital twin [177]
Certification of COVID-19 vaccination	Yolov5 deep learning model [181] Dense convolutional networks [182] Convolutional neural networks [183]	Ethereum [184-186] Smart contract [184,187-189] Hash algorithm [190] Blockchain adaptor (Canis Major) [191]	Mobile phone app [186] A digital Yellow Card on mobile phone [192]	N/A	Biometrics [181,193]
Postvaccination surveillance	N/A	Smart contract [194]	Mobile app (Respon) [195] Mobile app (vaxEffect@UniMiB) [196]	N/A	N/A

^a^N/A: not applicable.

#### Strategy of COVID-19 Vaccination

Nine articles [133-141] explored the applications of digital technologies in the strategy of COVID-19 vaccination. Machine learning algorithms were mainly applied to this field to help governments formulate better vaccination strategies in different scenarios. These digital solutions supported and complemented previous technologies, and the main barriers to their promotion lay in their data sources.

Four articles [135,136,138,139] tested COVID-19 mitigation strategies and the best vaccination criteria to minimize the number of infections and deaths. The remaining 5 studies [133,134,137,140,141] focused on priority groups and areas for COVID-19 vaccination. The unified Hierarchical Priority Classification-XGBoost model represented a significant improvement in predicting priorities for COVID-19 vaccination [133]. The Susceptible-Infected-Recovered model and a disease propagation graph simulated the optimum distribution of COVID-19 vaccines based on contact tracing data from cellular networks and Bluetooth signals [140]. Spatial artificial intelligence and satellite imagery were used to identify the location of vulnerable populations and target vaccination populations [141].

#### Distribution and Delivery of COVID-19 Vaccines

Twenty-two articles [142-163] involved the applications of digital technologies in the distribution and delivery of COVID-19 vaccines. Blockchain and algorithms were the leading technologies used in this field to optimize the distribution of COVID-19 vaccines. The prominent innovation feature was to support the existing vaccine distribution system, and the biggest promotion obstacles were blockchain-related technical issues, health care infrastructure issues, and data source issues.

The current platforms failed to meet the various storage and delivery conditions for different brands of COVID-19 vaccines [197-199], and blockchain was increasingly used to monitor the whole process from production to delivery, and to ensure vaccine safety in the supply chain. The blockchain applications in this field included Ethereum [155,156] and smart contracts [156-161], which could manage the data on COVID-19 vaccine distribution and automate traceability. The Ethereum solution was a decentralized application platform built on blockchain technology at a low cost, and smart contracts were automatically executable and code-based transactions on the blockchain, which were secure enough to avoid possible attacks and vulnerabilities [155]. Blockchain combined with unmanned aerial vehicle communication networks can ensure transparency of the vaccine supply chain and mitigate security attacks [157,161]. Additionally, 11 studies [142-152] utilized algorithms, such as LSTM, machine learning regression, and artificial neural networks, to design optimal vaccine distribution strategies, monitor vaccine storage temperature, and address supply chain issues.

#### Model Prediction of COVID-19 Vaccination

Six articles [164-169] applied algorithms in the model prediction of COVID-19 vaccination. Five of them focused on the prediction of vaccination progress and vaccination coverage from a specific country to the global context [165-169]. These algorithms mainly complemented and supported existing algorithms, with no obvious barriers to promotion. Besides the prediction of vaccine coverage, machine learning algorithms were used to analyze data from the vaccine adverse event reporting system to predict the safety of different COVID-19 vaccines across age groups [164].

#### COVID-19 Vaccination Services

Eleven articles [170-180] aimed to improve the efficiency and quality of COVID-19 vaccination services through algorithms and mHealth apps, as a support or complement to current vaccination services. Technical issues, such as operator difficulty in adopting these technologies, hindered the promotion of these technologies.

During the pandemic, drive-through clinics had been proposed as one of the effective approaches for temporary mass COVID-19 vaccination [200,201]. Machine learning models can help quickly assess the potential output of and design a smart parking system for drive-through vaccination clinics [170,172]. Digital technologies were also used to examine the accessibility of vaccine registration websites to ensure that the disabled can independently schedule vaccination appointments [179], to develop a multilingual app to facilize people with limited local language skills [174], to schedule people at more suitable vaccination centers [171], to remind about the next vaccination date [176], and to provide personalized emails/messages for vaccination promotion [178].

#### Certification of COVID-19 Vaccination

Thirteen articles [181-193] applied blockchain, mHealth, algorithms, and biometric technologies for COVID-19 vaccination certification. Eight of these studies were considered innovative, but some digital solutions had technical barriers, transaction costs, and ethical barriers to promotion. Many countries promoted vaccination certification to enable individuals to return to normal life [202,203]. The COVID-19 vaccination certificate built on blockchain technology had the advantages of decentralization, interoperability, security, transparency, and antitampering. Biometric technologies, such as face recognition [181,183] and iris recognition [193], were used to identify vaccination status, where deep learning algorithms like the Yolov5 model and convolutional neural networks can help perform this process. However, the promotion of biometric technologies may face ethical and data privacy issues. An artificial intelligence bot was also developed to detect fake vaccine certificates [182].

#### Postvaccination Surveillance

Three articles [194-196] focused on postvaccination surveillance. Two studies monitored adverse events following immunization through user-initiated reports in mobile apps (Respon and vaxEffect@UniMiB) [195,196], and another study established a dynamic monitoring model on COVID-19 vaccine effectiveness through health code blockchain [194]. As an innovative digital solution, technology realization was the main barrier to rollout.

## Discussion

### Principal Findings

Our review synthesized the global evidence on the applications of social media and digital technologies in COVID-19 vaccination. Social media has been applied to conduct sentiment/emotion analysis, topic analysis, behavioral analysis, dissemination and engagement analysis, and information quality analysis around COVID-19 vaccination. Sentiment/emotion analysis and topic analysis were the most dominant social media applications, while other applications were relatively few. Lexicon-based and machine learning approaches were developed to analyze massive textual data on social media. The development of digital technologies provided opportunities to determine the COVID-19 vaccination strategy, predict the vaccination process, optimize vaccine distribution and delivery, provide safe and transparent vaccination certificates, and perform postvaccination surveillance. The applied digital technologies included algorithms, blockchain, mHealth, IoT, and other technologies. Although these technologies have been successfully tried, there are still some barriers to their popularization.

We found that machine learning algorithms were widely applied in all 5 major COVID-19 vaccination fields, except postvaccination surveillance. Specifically, machine learning algorithms were applied to forecast the spread of the virus as well as the vaccination process [204], and an ensemble learning method with two or more learning algorithms could obtain better predictive performance than a single learning algorithm [205]. Blockchain was mainly used in vaccine distribution and vaccination certification, and mHealth, especially in the form of mobile apps, was a common technology in vaccination services, vaccination certification, and postvaccination surveillance. Blockchain technology was promoted owing to the security flaws and high costs of IoT [206,207]. As a blockchain platform, Ethereum [208] could execute smart contracts and be executed by all nodes in the peer-to-peer network. The Ethereum solution proposed by Musamih et al was generic and could be adapted to any type of vaccine tracing and monitoring program [155].

Although digital technologies had been applied to all aspects of COVID-19 vaccination and pandemic response [4], these digital predictions need to be verified for practicality in the real world. Digital technologies build a digital mirror of the real world to simulate the impact of various scenarios in virtual environments, which still need validation in the real world, namely digital twin [209]. Digital twin technology has been successfully used in many fields, including health care, and should also be used in vaccination [210,211]. Although the applications of digital technologies in addressing COVID-19 vaccination presented an irreversible trend and some technologies could be promoted for global adoption, most digital technologies still faced barriers to generalizability and scalability due to normative, legislative, ethical, or technical reasons [18]. For example, the adoption of blockchain involved technical issues, and the application of vaccination certification introduced ethical problems. Attention should be paid to legal and ethical issues when promoting these digital technologies.

Social media has been widely used to analyze public attitudes and behaviors during the COVID-19 pandemic [1], including vaccination attitudes and behaviors. In our review, most applications of social media in COVID-19 vaccination concentrated on content analysis, such as the sentiments expressed and the topics discussed on social media, but neglected the authenticity and reliability of relevant content in the context of “infodemic.” Social media analysis can help evaluate the information environment that the public is exposed to and its influences on vaccination. In the future, it is imperative to explore how to utilize social media platforms to intervene and increase the public’s willingness to undergo vaccination. Information released by authoritative institutions and professionals was generally of better quality than other sources, although there was limited evidence. The nature of social media contributes to celebrities and influencers having a tremendous amount of influence over what information is disseminated. More studies are warranted to assess the quality and reliability of information on social media [212] and how statements from the most influential people or institutions influence public attitudes toward vaccines.

During the COVID-19 epidemic, a considerable amount of COVID-19–related information is being spread through social media, resulting in an “infodemic” [213]. The endless stream of misinformation and rumors has led to negative public sentiments and irrational behaviors regarding the COVID-19 vaccine. Our review revealed that conversations about vaccine hesitancy were prevalent on social media, but tweets about vaccine advocacy and vaccine facts can improve public confidence. Moreover, information posted by authoritative social media users, such as governments and health professionals, can curb the spread of misinformation and consequently reduce vaccine hesitancy. In response to public concerns about vaccine safety and efficacy, and distrust in governments, the promotion of scientific data and the accuracy of the content on social media are critical to reduce negative public attitudes toward COVID-19.

The most used analysis techniques in social media studies were lexicon-based techniques and machine learning or deep learning techniques, which code and classify textual social media posts for analysis. A predefined lexicon can be used to code social media posts, and punctuation and negation need to be considered for its usage. TextBlob and VADER are 2 well-known lexicon-based techniques. Machine learning techniques can automatically analyze social media data by training classifiers through the annotation of a sampled data set, which significantly improves the accuracy and confidence of classification analysis. They have been increasingly used for social media analysis. The most used algorithms for machine learning were support vector machine and naïve Bayes, while the main deep learning algorithms were convolutional neural networks, recurrent neural networks, LSTM, and Bidirectional Encoder Representations from Transformers.

LDA topic modeling, an unsupervised machine learning algorithm, was widely used for clustering topics on social media. LDA has excellent performance in the traditional long text processing field, but its performance for short text is lacking [214]. As an unsupervised text classification algorithm based on the “bag-of-words model,” LDA may lead to the misclassification of short-text posts [215]. Nonnegative matrix factorization may produce higher-quality topics than LDA in short texts, and has been proven to be one of the most influential topic detection methods [216]. Furthermore, an improved Between Cluster-Balanced Iterative Reducing and Clustering using Hierarchies algorithm was proposed to reduce the number of classifications and provide a new model for topic discovery [214]. More effective algorithms are still needed for topic analysis in social media posts.

### Limitations

Our review has certain limitations. First, all included articles were in English, which may lead to limitations in the results. Second, social media users were skewed to young people, potentially disproportionately excluding older people or people with poor access to the internet, which may lead to bias when extrapolating the study results. Third, there may be some deficiencies in the models or algorithms in digital technical articles. For example, although the unsupervised clustering method allowed the classification of data quickly, the specific meaning of each cluster was unavailable and the reasons behind the clusters and exceptions were unclear. Finally, since COVID-19 is an emerging infectious disease and it takes time for studies to be published, there may exist more grey literature or preprint studies. Grey literature was not included in our review, which may lead to incomplete results.

### Conclusion

The applications of social media and digital technologies to address COVID-19 vaccination–related issues represent an irreversible trend. As a platform for public discourse, the prominent applications of social media were sentiment and topic analyses, and machine learning techniques were the most used technologies. It is warranted to review the accuracy and reliability of social media information and explore how to improve vaccination via social media. Digital technologies, such as machine learning algorithms and blockchain, have been widely applied to determine the COVID-19 vaccination strategy, predict the vaccination process, optimize vaccine distribution and delivery, provide safe and transparent vaccination certificates, and perform postvaccination surveillance. Attention should be paid to the ethical issues and health inequities arising from the digital divide while applying and promoting these technologies.

## Appendix

Multimedia Appendix 1 PRISMA-ScR (Preferred Reporting Items for Systematic Reviews and Meta-Analyses extension for Scoping Reviews) checklist.

Multimedia Appendix 2 Search strategy for the 6 peer-reviewed databases.

Multimedia Appendix 3 Scoring system for social media–based studies using the AXIS tool.

Multimedia Appendix 4 Characteristics, methods, and key findings of the included social media articles (n=114).

Multimedia Appendix 5 Characteristics, applications, innovation features, and generalizability of the included digital technology articles (n=64).

## Abbreviations

IoT: Internet of Things
LDA: latent Dirichlet allocation
LSTM: long short-term memory
mHealth: mobile health
PRISMA-ScR: Preferred Reporting Items for Systematic Reviews and Meta-Analyses extension for Scoping Reviews
VADER: Valence Aware Dictionary and Sentiment Reasoner
