# Supplementary material for: Applications of Social Media and Digital Technologies in COVID-19 Vaccination: Scoping Review
Source: J Med Internet Res. 2023 Feb 10;25:e40057. doi: 10.2196/40057 (PMC9924059; doi:10.2196/40057)
Supplement: Multimedia Appendix 2 [file jmir_v25i1e40057_app2.docx]

**Multimedia Appendix 2.** Search strategy for the 6 peer-reviewed databases.

| **Database** | **Step** | **Searching strategy** |
| --- | --- | --- |
| **PubMed** | #1 | "social media"[Mesh] OR "mass media"[Mesh] OR "social networking"[Mesh] OR "online social networking"[Mesh] OR "internet"[Mesh] OR "webcast" [Mesh] OR "blogging"[Mesh] OR "mobile applications"[Mesh] |
|  | #2 | "social media"[Title/Abstract] OR "mass media"[Title/Abstract] OR "social networking"[Title/Abstract] OR "social network"[Title/Abstract] OR internet[Title/Abstract] OR webcast[Title/Abstract] OR blogging[Title/Abstract] OR "online community"[Title/Abstract] OR "online post"[Title/Abstract] OR facebook[Title/Abstract] OR twitter[Title/Abstract] OR youtube[Title/Abstract] OR instagram[Title/Abstract] OR weibo[Title/Abstract] OR wechat[Title/Abstract] OR tiktok[Title/Abstract] OR line[Title/Abstract] OR reddit[Title/Abstract] OR whatsapp[Title/Abstract] OR telegram[Title/Abstract] OR "mobile application*"[Title/Abstract] OR "mobile app"[Title/Abstract] OR "chat bots"[Title/Abstract] |
|  | #3 | #1 OR #2 |
|  | #4 | "digital technology"[Mesh] OR "big data"[Mesh] OR "cloud computing"[Mesh] OR "blockchain"[Mesh] OR "artificial intelligence"[Mesh] OR "natural language processing"[Mesh] OR "deep learning"[Mesh] OR "machine learning"[Mesh] OR "neural networks, computer"[Mesh] OR "information technology"[Mesh] OR "internet of things"[Mesh] OR "crowdsourcing"[Mesh] OR "telemedicine"[Mesh] OR "telecommunications"[Mesh] OR "remote consultation"[Mesh] |
|  | #5 | digital[Title/Abstract] OR "digital health"[Title/Abstract] OR "digital technology"[Title/Abstract] OR "digital platform*"[Title/Abstract] OR "big data"[Title/Abstract] OR "data sharing"[Title/Abstract] OR "cloud computing"[Title/Abstract] OR "block chain"[Title/Abstract] OR "artificial intelligence"[Title/Abstract] OR AI[Title/Abstract] OR "natural language"[Title/Abstract] OR "deep learning"[Title/Abstract] OR "machine learning"[Title/Abstract] OR "neural network"[Title/Abstract] OR "information technology"[Title/Abstract] OR "internet of thing*"[Title/Abstract] OR IoT[Title/Abstract] OR crowdsourcing[Title/Abstract] OR telemedicine[Title/Abstract] OR telehealth[Title/Abstract] OR mhealth[Title/Abstract] OR "mobile health"[Title/Abstract] OR ehealth[Title/Abstract] OR telecommunication*[Title/Abstract] OR "remote consultation"[Title/Abstract] OR teleconsultation*[Title/Abstract] OR telesupport[Title/Abstract] OR telemonitoring[Title/Abstract |
|  | #6 | #4 OR #5 |
|  | #7 | #3 OR #6 |
|  | #8 | "COVID-19"[Mesh] OR "SARS-CoV-2"[Mesh]) OR "Coronavirus"[Mesh] |
|  | #9 | COVID-19[Title/Abstract] OR COVID[Title/Abstract] OR coronavirus[Title/Abstract] OR "coronavirus disease 2019"[Title/Abstract] OR 2019-nCov[Title/Abstract] OR "severe acute respiratory syndrome coronavirus 2"[Title/Abstract] OR sars-cov-2[Title/Abstract] |
|  | #10 | #8 OR #9 |
|  | #11 | "Vaccination"[Mesh] OR "Vaccines"[Mesh]) OR "Immunization"[Mesh] |
|  | #12 | vaccin*[Title/Abstract]) OR immunis*[Title/Abstract] OR immuniz*[Title/Abstract] |
|  | #13 | #11 OR #12 |
|  | #14 | #10 AND #13 |
|  | #15 | "COVID-19 Vaccines"[Mesh] |
|  | #16 | "COVID-19 vaccine*"[Title/Abstract] |
|  | #17 | #15 OR #16 |
|  | #18 | #14 OR #17 |
|  | #19 | #7 AND #18 |
|  | #20 | (7 AND 18) AND (("2019/12/01"[Date - Publication] : "2022/08/17"[Date - Publication])) Filters: English |
|  |  | Database: All accessible sub-databases |
| **Web of Science** | #1 | TS=("social media" OR "mass media" OR "social networking" OR "social network" OR internet OR webcast OR blogging OR "online community" OR "online post" OR facebook OR twitter OR youtube OR instagram OR weibo OR wechat OR tiktok OR line OR reddit OR whatsapp OR telegram OR "mobile application*" OR "mobile app" OR "chat bots") |
|  | #2 | TS=(digital OR "digital health" OR "digital technology" OR "digital platform*" OR "big data" OR "data sharing" OR "cloud computing" OR "block chain" OR "artificial intelligence" OR AI OR "natural language" OR "deep learning" OR "machine learning" OR "neural network" OR "information technology" OR "internet of thing*" OR IoT OR crowdsourcing OR telemedicine OR telehealth OR mhealth OR "mobile health" OR ehealth OR telecommunication* OR "remote consultation" OR teleconsultation* OR telesupport OR telemonitoring) |
|  | #3 | TS=("COVID-19" OR COVID OR coronavirus OR "coronavirus disease 2019" OR "2019-nCov" OR "severe acute respiratory syndrome coronavirus 2" OR "sars-cov-2") |
|  | #4 | TS=(vaccin* OR immunis* OR immuniz*) |
|  | #5 | TS=("COVID-19 vaccine*") |
|  | #6 | #1 OR #2 |
|  | #7 | #3 AND #4 |
|  | #8 | #5 OR #7 |
|  | #9 | #6 AND #8 |
|  | #10 | ((9 AND DOP=(2019-12-01/2022-08-17)) AND LA=(English) |
|  |  | Database: Core Collection database |
| **Embase** | #1 | exp social media/ or exp mass medium/ or exp social network/ or exp Internet/ or exp webcast/ or exp blogging/ or exp mobile application/ |
|  | #2 | ("social media" or "mass media" or "social networking" or "social network" or internet or webcast or blogging or "online community" or "online post" or facebook or twitter or youtube or instagram or weibo or wechat or tiktok or Line or reddit or whatsapp or telegram or "mobile application*" or "mobile app" or "chat bots").ti,ab,kw. |
|  | #3 | #1 OR #2 |
|  | #4 | exp big data/ or exp cloud computing/ or exp blockchain/ or exp artificial intelligence/ or exp natural language processing/ or exp deep learning/ or exp machine learning/ or exp artificial neural network/ or exp information technology/ or exp "internet of things"/ or exp crowdsourcing/ or exp telemedicine/ or exp telehealth/ or exp telecommunication/ or exp teleconsultation/ or exp telemonitoring/ |
|  | #5 | (digital or "digital health" or "digital technology" or "digital platform*" or "big data" or "data sharing" or "cloud computing" or "block chain" or "artificial intelligence" or {AI} or "natural language" or "deep learning" or "machine learning" or "neural network" or "information technology" or "internet of thing*" or {IoT} or crowdsourcing or telemedicine or telehealth or mhealth or "mobile health" or ehealth or telecommunication* or "remote consultation" or teleconsultation* or telesupport or telemonitoring).ti, ab, kw. |
|  | #6 | #4 OR #5 |
|  | #7 | #3 OR #6 |
|  | #8 | exp coronavirus disease 2019/ OR exp Severe acute respiratory syndrome coronavirus 2/ OR exp Coronavirus/ |
|  | #9 | ("COVID-19" or covid or coronavirus or "coronavirus disease 2019" or "2019-nCov" or "severe acute respiratory syndrome coronavirus 2" or "sars-cov-2").ti,ab,kw. |
|  | #10 | #8 OR #9 |
|  | #11 | exp vaccination/ OR exp vaccine/ OR exp immunization/ |
|  | #12 | (vaccin* or immunis* or immuniz*).ti,ab,kw. |
|  | #13 | #11 OR #12 |
|  | #14 | 10 AND #13 |
|  | #15 | exp SARS-CoV-2 vaccine/ |
|  | #16 | "COVID-19 vaccine*".ti,ab,kw. |
|  | #17 | #15 OR #16 |
|  | #18 | #14 OR #17 |
|  | #19 | #7 AND #18 |
|  | #20 | limit 19 to (english language and yr="2019 -Current") |
|  |  | Database: All accessible sub-databases |
| **EBSCO** | #1 | SU ("social media" OR "mass media" OR "social networking" OR "social network" OR internet OR webcast OR blogging OR "online community" OR "online post" OR facebook OR twitter OR youtube OR instagram OR weibo OR wechat OR tiktok OR line OR reddit OR whatsapp OR telegram OR "mobile application*" OR "mobile app" OR "chat bots") |
|  | #2 | SU(digital OR "digital health" OR "digital technology" OR "digital platform*" OR "big data" OR "data sharing" OR "cloud computing" OR "block chain" OR "artificial intelligence" OR AI OR "natural language" OR "deep learning" OR "machine learning" OR "neural network" OR "information technology" OR "internet of thing*" OR IoT OR crowdsourcing OR telemedicine OR telehealth OR mhealth OR "mobile health" OR ehealth OR telecommunication* OR "remote consultation" OR teleconsultation* OR telesupport OR telemonitoring) |
|  | #3 | SU(COVID-19 OR COVID OR coronavirus OR "coronavirus disease 2019" OR 2019-nCov OR "severe acute respiratory syndrome coronavirus 2" OR sars-cov-2) |
|  | #4 | SU(vaccin* OR immunis* OR immuniz*) |
|  | #5 | SU "COVID-19 vaccine" |
|  | #6 | (#3 AND #4) OR #5 |
|  | #4 | (#1 OR #2) AND #6  Limiters - Published Date: 20191201-20220831  Expanders - Apply equivalent subjects  Narrow by Language: - english  Search modes - Boolean/Phrase |
|  |  | Database: Academic Search Complete，Communication Source, CINAHL Complete, EconLit, MathSciNet via EBSCOhost，Library, Information Science & Technology Abstracts, OpenDissertations, MEDLINE |
| **Scopus** | #1 | TITLE-ABS-KEY ("social media" OR "mass media" OR "social networking" OR "social network" OR internet OR webcast OR blogging OR "online community" OR "online post" OR facebook OR twitter OR youtube OR instagram OR weibo OR wechat OR tiktok OR line OR reddit OR whatsapp OR telegram OR "mobile application*" OR "mobile app" OR "chat bots") |
|  | #2 | TITLE-ABS-KEY (digital OR "digital health" OR "digital technology" OR "digital platform*" OR "big data" OR "data sharing" OR "cloud computing" OR "block chain" OR "artificial intelligence" OR {AI} OR "natural language" OR "deep learning" OR "machine learning" OR "neural network" OR "information technology" OR "internet of thing*" OR {IoT} OR crowdsourcing OR telemedicine OR telehealth OR mhealth OR "mobile health" OR ehealth OR telecommunication* OR "remote consultation" OR teleconsultation* OR telesupport OR telemonitoring) |
|  | #3 | TITLE-ABS-KEY ("COVID-19" OR covid OR coronavirus OR "coronavirus disease 2019" OR "2019-nCov" OR "severe acute respiratory syndrome coronavirus 2" OR "sars-cov-2") |
|  | #4 | TITLE-ABS-KEY (vaccin* OR immunis* OR immuniz*) |
|  | #5 | TITLE-ABS-KEY ("COVID-19 vaccine*") |
|  | #6 | #1 OR #2 |
|  | #7 | #3 AND #4 |
|  | #8 | #5 OR #7 |
|  | #9 | #6 AND #8 |
|  | #10 | PUBYEAR>2018 AND (LIMIT-TO (LANGUAGE, "English")) |
|  | #11 | #9 AND #10 |
|  |  | Database: All accessible sub-databases |
| **IEEE Xplore** | #1 | "All Metadata": ("social media" OR "mass media" OR "social networking" OR "social network" OR internet OR webcast OR blogging OR "online community" OR "online post" OR facebook OR twitter OR youtube OR instagram OR weibo OR wechat OR tiktok OR line OR reddit OR whatsapp OR telegram OR "mobile application*" OR "mobile app" OR "chat bots") |
|  | #2 | "All Metadata": (digital OR "digital health" OR "digital technology" OR "digital platform*" OR "big data" OR "data sharing" OR "cloud computing" OR "block chain" OR "artificial intelligence" OR AI OR "natural language" OR "deep learning" OR "machine learning" OR "neural network" OR "information technology" OR "internet of thing*" OR IoT OR crowdsourcing OR telemedicine OR telehealth OR mHealth OR "mobile health" OR ehealth OR telecommunication* OR "remote consultation" OR telesupport OR teleconsultation* OR telemonitoring) |
|  | #3 | "All Metadata": (COVID-19 OR COVID OR coronavirus OR "coronavirus disease 2019" OR 2019-nCov OR "severe acute respiratory syndrome coronavirus 2" OR sars-cov-2) |
|  | #4 | "All Metadata": (vaccin* OR immunis* OR immuniz*) |
|  | #5 | #3 AND #4 |
|  | #6 | "All Metadata": ("COVID-19 vaccin*") |
|  | #7 | #5 OR #6 |
|  | #8 | (#1 OR #2) AND #7 AND 2019.12.1-2022.8.17/ENGLISH |
|  |  | Database: All accessible sub-databases |
