# Supplementary material for: Applications of Social Media and Digital Technologies in COVID-19 Vaccination: Scoping Review
Source: J Med Internet Res. 2023 Feb 10;25:e40057. doi: 10.2196/40057 (PMC9924059; doi:10.2196/40057)
Supplement: Multimedia Appendix 3 [file jmir_v25i1e40057_app3.docx]

**Multimedia Appendix 3.** Scoring system for social media–based studies using the AXIS tool.

| Appraisal items | | Max score ^a^ | Individual score | |
| --- | --- | --- | --- | --- |
|  |  |  | **0** | **1** |
| Introduction | | | | |
| 1 | The objective(s) of the study was clear | 1 | No/Don’t know | Yes |
| Methods | | | | |
| 2 | The study design for the stated objective(s) as appropriate | 1 | No/Don’t know | Yes |
| 3 | The sample size was justified | NA | - | - |
| 4 | Clear criteria for the inclusion of posts | 1 | No/Don’t know | Yes |
| 5 | The sample frame was taken from an appropriate population base | 1 | No/Don’t know | Yes |
| 6 | The selection process was likely to select participants that were representative of the target population | NA | - | - |
| 7 | Measures were undertaken to address and categorize non-responders | NA | - | - |
| 8 | The risk factor and outcome variables were measured appropriately | 1 | No/Don’t know | Yes |
| 9 | The risk factor and outcome variables were measured correctly using instruments that had been trialed, piloted, or published previously | 1 | No/Don’t know | Yes ^b^ |
| 10 | The determinant of statistical significance and/or precision estimates is clear | 1 | No/Don’t know | Yes |
| 11 | The methods were sufficiently described to enable them to be repeated | 1 | No/Don’t know | Yes |
| Results | | | | |
| 12 | The basic data was adequately described | 1 | No/Don’t know | Yes |
| 13 | The response rate raise does not concern non-response bias | NA | - | - |
| 14 | The information about non-responders described | NA | - | - |
| 15 | The results were internally consistent | 1 | No/Don’t know | Yes |
| 16 | The results of the analyses described in the methods were presented | 1 | No/Don’t know | Yes |
| Discussion | | | | |
| 17 | The authors’ discussions and conclusions were justified by the results | 1 | No/Don’t know | Yes |
| 18 | The limitations of the study were discussed | 1 | No/Don’t know | Yes |
| Others | | | | |
| 19 | No existence of funding sources or conflicts of interest | 1 | No/Don’t know | Yes |
| 20 | Ethical approval or consent of participants was attained | NA | - | - |
|  | Total | 15 | | |

Note. a: “low risk” is from 14 to 12, “some concern” is from 11 to 10, and “high risk” is 8 and below.

b: using the published methods/codebook for coding or Cohen's kappa coefficient to measure inter-rater reliability, or with a pilot study.
