# Supplementary material for: Applications of Social Media and Digital Technologies in COVID-19 Vaccination: Scoping Review
Source: J Med Internet Res. 2023 Feb 10;25:e40057. doi: 10.2196/40057 (PMC9924059; doi:10.2196/40057)
Supplement: Multimedia Appendix 4 [file jmir_v25i1e40057_app4.docx]

**Multimedia Appendix 4.** Characteristics, methods, and key findings of the included social media articles (n=114).

| Reference | Study period | Country | Social media | Method (technology) | Key findings |
| --- | --- | --- | --- | --- | --- |
| **Sentiment/emotion analysis (n=70)** | | | | | |
| ***Lexicon-based approaches (n=40)*** | | | | | |
| Mir et al | 1/12-2/13,2021 | / | Twitter | Lexicon-based approaches (VADER) | A gradual decline in the number of tweets is observed over time. The public remains positive and hopeful about COVID-19 vaccines. |
| Sutrave et al | 7/1,2020-1/28,2021 | / | Twitter | National Research Council Sentiment Lexicon based approaches (syuzhet) | The public has positive attributes and outlooks about COVID-19 vaccines. Most of them expressed positive emotions of trust and anticipation, indicating confidence in health experts and vaccines. |
| Claudia et al | 3/15-4/14,2021 | / | Twitter | National Research Council Sentiment Lexicon based approaches (syuzhet) | The infodemic primarily relies on strong negative emotions. Vaccine sentiment is influenced by real-time news and other information circulating on the Internet. Negative tweets concern vaccine unsafety or vaccine skepticism in general, attacks on the pharmaceutical industry/institutions/politicians, and criticism regarding the suspension of AstraZeneca. |
| Lyu et al | 9/28,2020-11/4,2020 | US | Twitter | Lexicon-based approaches (VADER) | 58% pro-vaccine, 19% vaccine-hesitant, with the rest anti-vaccine. Socioeconomically disadvantaged groups are more likely to hold polarized opinions on COVID-19 vaccines. People with bad experience during the pandemic are more likely to keep anti-vaccine opinions. |
| Lyu et al | 3/11,2020-1/31,2021 | / | Twitter | National Research Council Sentiment Lexicon based approaches (syuzhet) | The weekly mean sentiment was increasingly positive in general. Emotion analysis showed that trust was the most predominant emotion, followed by anticipation, fear, sadness, etc. |
| Marcec et al | 12/1,2020-3/31,2021 | / | Twitter | The AFINN lexicon-based approaches | While sentiments regarding Pfizer and Moderna vaccines appeared positive and stable, sentiments regarding the AstraZeneca/Oxford vaccine became negative. |
| Melton et al | 12/1,2020-5/15,2021 | / | Reddit | Lexicon-based approaches (TextBlob) | Public sentiment toward COVID-19 vaccines had been primarily positive: 56.68% of the posts were positive, 27.69% negative, and 15.63% neutral. |
| Monselise et al | 12/16,2020-2/13,2021 | US | Twitter | Lexicon based approaches (VADER) | 46.9% of tweets were negative, with emotions of mostly fear, followed by sadness and anger; 33.2% of tweets were positive, with emotions of joy and hopefulness. And 19.9% of tweets were neutral. |
| Mudassir et al | 2/22,2021-3/7,2021 | India | Twitter | Lexicon based approaches (The ABSA classifier) | The ABSA model is better than TextBlob and VADER. The vaccination of the Prime Minister of India caused a sudden spike in positive tweets. |
| Mushtaq et al | 12/12,2020-10/24,2021 | / | Twitter | Lexicon based approaches (VADER) | Public sentiments about Covaxin are more positive than Moderna, Pfizer, and Sinopharm. |
| Ong et al | 1,2022-2,2022 | Malaysia | Twitter | Lexicon based approaches (SentiWordNet model) | Nearly half of all tweets expressed a positive public opinion about the vaccine booster, around one-third were negative, and less than 20% were neutral. |
| Akcan et al | 3/115,2021-4/15,2021 | Turkey | Twitter | National Research Council Sentiment Lexicon based approaches | Users' attitudes towards the BioNTech brand were neutral, and the brand was perceived to be more trustworthy than the Sinovac brand in the comments shared. |
| Gottipati et a | 1,2020-3,2021 | / | Twitter | Lexicon based approaches (VADER) | Negative sentiment was higher at the pandemic declaration and gradually changed to the positive sentiment with the COVID-19 vaccine development/rollout. |
| Gulati | 1-5,2021 | / | Twitter | National Research Council Sentiment Lexicon based approaches | The dominance of positive sentiments is double its negative counterpart. The highest sentiment is "trust," followed by "fear." The least visible sentiment is "surprise." |
| Liu et al | 11/1,2020-1/31,2021 | / | Twitter | Lexicon based approaches (VADER) | Tweets with positive, neutral, and negative sentiments were 42.8%, 26.9%, and 30.3%, respectively. Public sentiment on COVID-19 vaccines varied significantly over time and geography. |
| Li et al | 1/15,2020-9/30,2021 | China | Weibo | Lexicon based approaches (the SnowNLP) | From the temporal perspective, public emotion declines in the later stage, but overall emotional performance is positive and stabilizing. This decline in emotion is mainly associated with ambiguous information about the COVID-19 vaccine. |
| Shah et al | 9,2020-3,2021 | / | Twitter | Lexicon based approaches (SentiStrength) | From September 2020 to March 2021, 27 % of user were more confident in the safety and effectiveness of the vaccine. |
| Stella et al | 10/12,2020- 3/19,2021 | / | Twitter | Lexicon-based approaches | The negative, inhibiting emotions such as disgust can amplify content sharing while sadness inhibits endorsement of online posts. |
| Feizollah et al | 1/1,2020-4/30,2021 | / | Facebook, Twitter | Lexicon based approaches (VADER) | The sentiment toward halal vaccine is primarily neutral in Twitter data, whereas it is positive in Facebook data. Trust is the most present emotion, followed by anticipation and fear. |
| Bari et al | 3/12,2020-7/20,2021 | US | Twitter | Lexicon based approaches (TextBlob) | Across US geographic regions, there is a correlation between positive sentiment and increased vaccine uptake. |
| Alam et al | 12/21,2020-7/21,2021 | / | Twitter | Lexicon based approaches (VADER) | Sentiments fluctuated over time. Neutral tweets formed the majority, and negative reactions were less frequent. |
| Daradkeh et a | 1/1-3/31,2021 | / | Twitter | Lexicon based approaches (VADER) | The proportion of tweets with negative sentiment in the replies was higher for misinformation than for factually correct information. Misinformation with negative sentiment is more likely to be re-posted and shared than misinformation with positive sentiment, with increased audience engagement and interaction. |
| Ali et al | 2/1-3/31,2021 | US | Twitter | Lexicon based approaches (VADER and TextBlob) | The share of positive sentiment tweets decreased over time. The percentage of tweets with positive sentiment was higher compared to the rate of tweets with negative sentiment. |
| Bi et al | 12,2020-4,2021 | China | Douban | Lexicon based approaches | The attitude of netizens changed from a more negative attitude to a more positive attitude with the development of events, and their attitudes fluctuated until they finally tended to be more positive. |
| Chandrasekaran et al | 1/1,2020-4/30,2021 | / | Twitter | Lexicon based approaches (VADER) | The proportion of positive or overly positive tweets was always more significant than negative or highly negative tweets. |
| Gaweł et al | 3-10,2021 | Global | Twitter | Lexicon based approaches (TextBlob, Flair NLP, Stanza, and NLTK VADER) | NLTK VADER analyzed the public sentiment most correctly than the other algorithms. The opinion of most folks regarding the vaccination drive on Twitter was positive. |
| Greyling et al | 2/1-7/31,2021 | South Africa, New Zealand, Australia, seven Northern hemisphere countries | Twitter | National Research Council Sentiment Lexicon based approaches | Precise information about the COVID-19 vaccines, also disseminated via social media, can increase positivity towards the COVID-19 vaccine. |
| Huangfu et al | 12/14,2020-4/30,2021 | / | Twitter | Lexicon based approaches (VADER) | Most sentiments toward COVID-19 vaccines were neutral and positive. Positive sentiment was stronger than negative sentiment throughout the period. The main topic in the positive and neutral domain was encouraging people to get vaccinated. |
| Karami et al | 11/1,2020-2/28,2021 | US | Twitter | Lexicon based approaches (Brandwatch) | The negative and non-negative sentiments of tweets containing terms related to the COVID-19 vaccine had decreasing and increasing trends, respectively. |
| Luo et al | 12/1,2020-2/20,2021 | US and China | Twitter，Weibo | Lexicon based approaches (The LIWC, TextMind software) | Twitter users were prone to disclose individual vaccination experiences and express anti-vaccine attitudes. In comparison, Weibo users manifested evident deference to authorities and exhibited more positive feelings toward the COVID-19 vaccine. |
| Liew et al | 11-week period after 11/18,2020 | / | Twitter | Lexicon based approaches (VADER) | Tweets with negative sentiments largely fell within the themes of emotional reactions and public concerns related to COVID-19 vaccines. |
| Wang et al | 3,2020-3,2021 | / | Twitter | National Research Council Sentiment Lexicon based approaches | Discourses about COVID-19 vaccines contain more positive sentiment than negative sentiment. Trust is the most salient emotion associated with COVID-19 vaccine discourses, followed by anticipation, fear, joy, sadness, anger, surprise, and disgust. |
| Xie et al | 3/5,2020-1/25,2021 | US | Twitter | Lexicon based approaches (VADER) | There were more positive tweets than negative tweets. The vaccine development and the pandemic influenced public discussion and perception of COVID-19 vaccines on Twitter. |
| Zhang et al | 10/18,2020-5/15,2021 | China | Weibo | Lexicon based approaches (SnowNLP) | The positivity toward COVID-19 vaccines in China tends to fluctuate from 45.7% to 77.0% and is intuitively correlated with public health events. |
| Zhang et al | 6/9,2020-7/31,2021 | / | Twitter | Lexicon based approaches (VADER) | The sentiments gradually stabilized and tended to relatively positive levels at the beginning of vaccination, but after June 2021, they all had a considerably downward trend. The population had lower sentiments and more fear toward vaccines than general topics. |
| Kwok et al | 1/22,2020 to 10/20,2020 | Australia | Twitter | National Research Council Sentiment Lexicon based approaches | Nearly two-thirds of the sentiments of all tweets expressed a positive public opinion about the COVID-19 vaccine. Trust and anticipation were the prominent positive emotions, while fear was the top negative emotion. |
| Shim et al | 2/23,2021 to 3/22,2021 | Korea | Twitter | The KNU Korean Sentiment Lexicon based approaches (Bi-LSTM model) | The ratio of positive and negative tweets was similar immediately before and after the commencement of vaccinations. Still, negative tweets were prominent after the increase in confirmed COVID-19 cases. |
| Praveen et al | 9,2020 to 12,2020 | India | Twitter | Lexicon based approaches (TextBlob) | There is a strong correlation between the increase in positive sentiments toward the COVID-19 vaccine and the number of COVID-19 cases. |
| Yousefinaghani et al | 1/7,2020-1/3,2021 | / | Twitter | Lexicon based approaches (VADER) | Positive being the dominant polarity and having higher engagements. Positive hashtags were mainly related to medical research and health services, while negative hashtags were related to commentary on political figures and conspiracy theories. |
| Hu et al | 3/1,2020-2/28,2021 | US | Twitter | National Research Council Sentiment Lexicon based approaches | Trust was the dominant emotion towards vaccination over the full timeline of the pandemic. It was followed by anticipation, fear, sadness, anger, joy, disgust, and surprise. |
| ***Machine learning approaches (n=19)*** | | | | | |
| Sufi et al | 6/15,2021-12/31,2021 | / | Twitter | Machine learning algorithms (Microsoft Azure Cognitive services) | Anti-Vax social movement-related tweets demonstrate 72% higher negativity than the global average negative sentiment of 0.46. Pro-Vax-related tweets also showed a 65% higher level of negativity. |
| Cathy et al | 1/13,2021 | Canada | Reddit and Twitter | Machine learning algorithms (Random Forest regression model) | Sentiments toward vaccine uptake significantly differed across cities. Vaccine-related comments expressed significantly higher positive sentiments. |
| Delcea et al | 7/12-8/11,2021 | / | Twitter | Machine learning algorithms (MNB, RF and SVM) | The number of neutral tweets exceeded those in favor and against tweets in both the cleaned and the entire datasets. |
| Mourad et al | 2/1,2020-4/30,2020 | Arab region | Twitter | Deep learning algorithms (the XLM-T model) | Positive sentiment increased over time, with some countries having predominantly positive sentiment compared to negative or neutral ones, such as Saudi Arabia, Kuwait, Bahrain, and Jordan. |
| Nezhad et al | 4/1,2021-9/30,2021 | Iranian | Twitter | Hybrid deep-learning model (CNN-LSTM and Word2vec) | The issue of a mandatory homegrown vaccine led directly to negative sentiments toward it. In contrast, reports of an import ban on foreign vaccines caused positive sentiments toward them and further negative sentiments toward the homegrown vaccine. |
| Niu et al | 8/1,2020-6/30,2021 | Japan | Twitter | Machine learning algorithms (AWS) | The sentiments of most tweets were neutral, with negative sentiment exceeding positive sentiment in volume. |
| Pan et al | 8,2020-8,2021 | / | Twitter | Deep learning algorithms (BiLSTM) | Public attitude toward the COVID-19 vaccine fluctuated, mainly influenced by the timing of relevant news releases and the fact that most people supported the COVID-19 vaccine. |
| Portelli et al | 12/10,2020-9/7,2021 | / | Twitter | Deep learning algorithms (RoBERTa model) | The general sentiment of Twitter users toward vaccines is negative/neutral. |
| Rahmanti et al | 10/15,2020-4/12,2021 | Indonesia | Twitter | Machine learning algorithms (Naïve Bayes) | Public sentiment on the COVID-19 vaccine is correlated with the increase in vaccination coverage. The COVID-19 vaccine sentiment trend gradually changes to positive with the growth of trust emotion. |
| Shahriar et al | 12/12,2020-11/24,2021 | / | Twitter | Deep learning algorithms (RNN, CNN, GRU, BiLSTM, and LSTM) | The people's positive attitudes are higher than negative attitudes toward vaccine brands. The positive attitude of people towards Covaxin is higher than other vaccine brands. |
| Aygun et al | 11/15,2020-3/15,2021 | USA, UK, Canada, Turkey, France, Germany, Spain, Italy | Twitter | Deep learning algorithms (BERT) | Positive tweets increased with vaccination studies in countries in general. Politics and health were the most frequently addressed topics in tweets that spoke negatively about vaccination. |
| Khan et al | 8/10,2020-4/4,2021 | Saudi Arabia | Twitter | Machine learning algorithms (SVM, NB, LR, LSTM) | People in KSA are usually either pro-vaccination or neutral. LSTM outperformed the ML models with an accuracy, recall, and F1 score of 0.95 and a precision of 0.96. |
| Lee et al | 1,2020-8,2021 | / | Twitter | Deep learning algorithms (BERT) | The balanced emotion model shows that fear is the most dominant emotion. The extended emotion model shows various negative emotions such as panic, fear, and shame as the dominant emotions in the tweet hashtag groups such as COVID-19, vaccines, and anti-vaxxers. |
| Jang et al | 12,2020-5,2021 | Canada | Twitter | Machine learning algorithms (ABSApp) | Compared to the sentiment of the remaining tweets, the most retweeted or liked tweets showed more positive sentiment overall toward crucial aspects, especially vaccines and vaccination. |
| Jun et al | 11/1,2020-8/15,2021 | 192 countries | Twitter | Deep learning algorithms (Brandwatch’s sentiment analysis tool) | The negative sentiment appeared 1.90 times more frequently than the positive sentiment. Fear, anger, or sadness appeared 0.70 times less regularly than joy. |
| Xue et al | 1/1,2020-4/30,2021 | US | Facebook | Machine learning algorithms (Google Cloud Natural Language AI and IBM Watson Tone Analyzer) | Third-party fact-checkers tended to evoke heightened comment anger, whereas comments on posts from health media and hospitals expressed less negative emotion. |
| Wang et al | 1/23,2020-2/11,2021 | China | Weibo | Machine learning algorithms (Baidu's AipNLP) | There was more information supporting COVID-19 vaccination on Weibo than against vaccination. Anti-vaccine tweets were more popular than pro-vaccine tweets but not significant. |
| Villavicencio et al | 3/1,-3/31,2021 | Philippines | Twitter | Machine learning algorithms (Naïve Bayes) | The majority of the tweets in the Philippines were positive and enthusiastic about the idea of vaccination, while 9% had neutral and 8% had negative sentiments. |
| Amanatidis et al | 12/1-12/27,2020 | Greece | Instagram | Deep learning algorithms (CNN) | Posts made for #pfizer seem more positive than the other two hashtags. |
| ***Manual coding (n=4)*** | | | | | |
| Al-Zaman et al | 3/8-12/2,2020 | Bangladesh | Facebook | Manual coding | Users’ reaction to the vaccine issue is dominantly positive, though they may show a highly negative attitude toward vaccine misinformation. |
| Olszowski et al | 7/26-12/11,2021 | Poland | Twitter | Manual coding | Approximately 60% of the analyzed content belongs to Category A (against mandatory vaccination). |
| Wang et al | 12/2,2020-1/30,2021 | global |  | Manual coding | About half of the tweets used a positive tone, nearly half used a neutral tone, and only 3.1% used a negative tone. |
| Wang et al | 12/23,2021 | / | TikTok | Manual coding | Joy was the most frequently expressed emotion in videos that supported COVID-19 vaccination. Sadness was the least-mentioned emotion. |
| ***hybrid methods (n=7)*** | | | | | |
| Niu et al | 2/1-9/30,2021 | Japan | Twitter | AWS; National Research Council Sentiment Lexicon | Negative sentiment overwhelmed positive sentiment, and fear was the dominant emotion across the period. |
| Reshi et al | / | Global | Twitter | Lexicon-based approaches (TextBlob，VADER，AFINN); Machine learning algorithms (DT, RF，LR, CNN, LSTM, RNN, GRU, CNN-LSTM, LSTM-GRNN) | The positive sentiment ratio is higher than negative sentiments in tweets related to COVID-19 vaccinations. LSTM-GRNN performs significantly better than all the machine learning and deep learning models used in this study. |
| Roe et al | 7/1-7/21,2021 | UK | Twitter | Lexicon-based approaches (VADER); Machine learning algorithms (Microsoft Azure) | The majority of tweets were found to be negative in sentiment, followed by positive and neutral. The negative tweets displayed a higher intensity of sentiment than the positive tweets. |
| Ansari et al | 5/15-6/25,2021 | Global | Twitter | Lexicon-based approaches (TextBlob); Machine learning algorithms (NB) | Overall, Tweets were generally negative in tone. |
| Cepeda et al | 1,2020-8,2021 | inIreland | Twitter | Lexicon-based approaches (VADER); Machine learning algorithms (SVM) | Negative feelings were predominated in Ireland, although there was no significant difference compared to positive tweets. The Pfizer and Moderna vaccines showed a positive feeling, and the AstraZeneca and J&J vaccines had a negative sentiment. |
| Gao et al | 12/5,2020-1/7,2021 | China | weibo | Lexicon-based approaches (bag-of-words model); Machine learning algorithms (CNN) | "Good" was the most increased positive emotion and indicated great public appreciation for the production capacity and free vaccination. "Fear" was the significantly increased negative emotion and reflected the public concern about the safety of the vaccines. |
| Hussain et al | 3/1-11/22,2020 | UK and US |  | VADER, TextBlob, and BERT | Averaged public sentiment toward COVID-19 vaccines has been mostly positive and similar in both the United Kingdom and the United States across both platforms. |
| **Topic analysis (n=53)** | | | | | |
| ***Latent Dirichlet Allocation (n=24)*** | | | | | |
| Jiang et al | 2/20-3/31,2020 | US | Twitter | Latent Dirichlet Allocation (LDA) | Anti-vaccination arguments and conspiracy theories were major sources of vaccination opposition. Conspiracy tweets were more likely to be posted by users with many followers. Changes in topics over time were closely related to news or events related to vaccine development. |
| Kruttika et al | 7/1,2020-1/28,2021 | / | Twitter | LDA | Those who show positive opinions appreciate the efforts of leadership, medical experts, and pharmaceutical companies in developing the COVID-19 vaccine. Many people expressed negative opinions mainly because of their disbelief in the government, doubts about the efficacy of the vaccines, and concerns about adverse reactions. |
| Cathy et al | 7/13,2020-6/14,2021 | Canada | Reddit and Twitter | LDA | When case numbers increase, more discussion is generated. Despite feeling generally positive about vaccines, there is still apprehension. |
| Camelia et al | 7/12-8/11,2021 | / | Twitter | LDA | Most of the discussion topics included side effects, the existence of alternatives, hiding relevant information, mistrust, and scam. |
| Lyu et al | 3/11,2020-1/31,2021 | / | Twitter | LDA | Opinions about vaccination were the most tweeted topic. Major events about COVID-19 vaccines primarily drove public COVID-19 vaccine-related discussion on Twitter. |
| Ma et al | 1/1-3/14,2021 | / | Twitter | LDA | Common reasons for vaccine hesitation include concerns about safety, skepticism about the development and approval of the vaccines, vaccine efficacy, and judgments of personal risk from COVID infection. |
| Melton et al | 12/1,2020-5/15,2021 | / | Reddit | LDA | Community members mainly focused on side effects rather than outlandish conspiracy theories. Due to the severity of some documented side effects and their wide media coverage, it is highly conceivable that side effects are a major contributor to hesitancy. |
| Niu et al | 8/1,2020-6/30,2021 | Japan | Twitter | LDA | The discussion of all three vaccines mainly concerned effectiveness and side effects. The public tended to focus on the effectiveness of Pfizer in preventing infection, as opposed to Moderna, which tended to focus more on its efficacy against mutated viruses and its mRNA development technology. |
| Gottipati et al | 1,2020-3,2021 | / | Twitter | LDA | When the vaccine rollout began, the dominant topics were Appointments and Reactions. |
| Li et al | 1/15,2020-9/30,2021 | China | Weibo | LDA | The dissemination of specific information, the local epidemic prevention and control status, the research progress of vaccines, and the vaccination schedule may be potential drivers of topic evolution in local regions. The critical social events or statements issued by the authorities potentially impact the public discussion volume of the COVID-19 vaccine. |
| Shah et al | 9,2020-3,2021 | / | Twitter | LDA | After the vaccine rollout, most users from G1 than G2 were more optimistic about the fight against COVID-19. |
| Cotfas et al | 12/8,2020-1/7,2021 | UK | Twitter | LDA | The variation in the number of tweets posted connected with the major events reported by the news in the corresponding days. Mistrust and side effects were the most encountered topics related to vaccine hesitancy. |
| Daradkeh et al | 1/1-3/31,2021 | / | Twitter | LDA | Authoritative and reliable information disseminators such as government agencies, major media outlets, and key opinion leaders play a massively influential role in polarizing opinions, which can amplify (or contain) the spread of misinformation among target audiences. |
| Ginossar et al | 2/1-6/23,2021 | Global | YouTube and Twitter | LDA | Content analysis of the 20 most tweeted videos revealed that the majority opposed vaccination and included conspiracy theories. The most tweeted topic over time framed vaccinations as conspiracies that aimed to infringe on individual freedom and civil rights. |
| Karami et al | 11/1,2020-2/28,2021 | US | Twitter | LDA | The vaccine sites and the vaccination and election topics were the most and least popular topics. There was high discussion on topics related to vaccination hesitancy and immunity. |
| Wang et al | 3,2020-3,2021 | / | Twitter | LDA | Over time, tweets about vaccine advocacy and facts have become more dominant. In contrast, tweets about vaccine hesitancy have become less dominant among COVID-19 vaccine discourses, suggesting that the public has become more confident about COVID-19 vaccines as they obtain more information. |
| Xie et al | 3/5,2020-1/25,2021 | US | Twitter | LDA | The development of COVID-19 vaccines and the severity of the COVID-19 pandemic could significantly influence the discussion of COVID-19 vaccines on Twitter. |
| Zhang et al | 1/1,2020-4/30,2021 | US | Twitter | LDA | The tweets were clustered around vaccine access, followed closely by vaccine efficacy and rollout, vaccine development, and people s views. |
| Guntuku et al | 12/1,2020-2/28,2021 | US | Twitter | LDA | The Twitter discourse around COVID-19 vaccines in the United States varied significantly across different communities and changed over time. |
| Kwok et al | 1/22-10/20,2020 | Australia | Twitter | LDA | Some had misconceptions and complaints about COVID-19 and infection control measures, while others advocated for pharmaceutical and non-pharmacological measures against COVID-19. |
| Shim et al | 2/23-3/22,2021 | Korea | Twitter | LDA | The most frequently tweeted topic was vaccine hesitation, consisting of fear, flu, the safety of vaccination, time course, and degree of symptoms. |
| Praveen,et al | 9-12,2020 | India | Twitter | LDA | Fear of health and allergic reactions to the vaccine are the two prominent issues that concern Indian citizens regarding the COVID-19 vaccine. A considerable percentage of the Indian population on social media does not trust the government and pharma companies. |
| Hu et al | 3/1,2020-2/28,2021 | US | Twitter | LDA | Compared to critical events in the development of COVID-19 vaccines, comments from public figures on vaccination could trigger bigger changes in public sentiment. |
| Liu et al | 11/1,2020-1/31,2021 | / | Twitter | LDA | The prevalence of tweets containing positive behavioral intentions increased over time. |
| ***Manual coding (n=17)*** | | | | | |
| Laforet et al | 2/17,2021 | / | YouTube | Manual coding | Consumer videos also mentioned anti-vaccination sentiment, fear, or distrust of the vaccines more often than medical professional videos and television or internet-based news videos. |
| Bonnici et al | 4/30-5/4,2019 | / | Facebook | Manual coding | AVP dominated the conversation, with nearly 60 percent of posts representing their engagement. |
| Ngai et al | 9/15,2019-8/16,2021 | Poland | Facebook | Manual coding | The most common content themes disseminated in COVID-19 antivaccine misinformation on Facebook were safety concerns, followed by conspiracy theories. A conversational style and format and language features that mimicked news media and scientific reports were frequently used to spread antivaccine misinformation. |
| Baines et al | 11/20,2020-1/6,2021 | US | Parler | Manual coding | Parler users discuss several reasons for vaccine refusal and skepticism regarding the vaccine's efficacy, refusal among healthcare workers to get vaccinated, and potential side effects. |
| Fieselmann et al | 1/20,2020-5/2,2021 | Germany | Instagram, Twitter, and YouTube | Manual coding | Six main reasons for refusing a COVID-19 vaccination were identified: Low perceived benefit of vaccination, low perceived risk of contracting COVID-19, health concerns, lack of information, systemic mistrust, and spiritual or religious reasons. |
| Cruickshank et al | 2/1-6/30,2020 | / | Twitter | Manual coding | The content of these websites demonstrated the politicization of vaccination. |
| Gao et al | 7/23-7/26,2021 | China | Weibo | Manual coding | Weibo users were prone to showing respect for the authorities and a more positive attitude toward COVID-19 vaccines. External sources outside of the vaccine might cause vaccine hesitancy, such as distrust in the media. |
| Hernández-García et al | 2/9,2021 | Spanish | YouTube | Manual coding | The most discussed topics were target groups for vaccination and safety. Compared to those of media, videos made by health professionals showed a greater positive tone. |
| Khan et al | 5/24-6/17,2021 | / | Twitter | Manual coding | Most tweeters had a favorable attitude toward the COVID-19 passports. Unfavorable attitudes toward the COVID-19 passport were based on reasons such as a lack of common standard or consensus and personal freedoms & human rights. |
| Küçükali et al | 12/9,2020-1/8,2021 | Turkey | Twitter | Manual coding | We found that 22.0% of the tweets included at least one anti-vaccination theme. Poor scientific process was the most predominant theme, followed by conspiracy theories and suspicion towards manufacturers. |
| Li et al | 8/9,2020-4/18,2021 | / | Tw itter | Manual coding | The national level showed that the VAI moved from negative to positive in 2020 and maintained steady after January 2021. |
| Wang et al | 12/23,2021 | / | TikTok | Manual coding | Confidence was the most frequently expressed belief on TikTok and was positively related to the number of comments that supported the video. Emphasizing the safety and effectiveness of vaccination is the most helpful to promote vaccines. |
| Wawrzuta et al | 8/1,2021-2/1,2022 | Poland | Facebook, Twitter, Instagram, and TikTok | Manual coding | The anti-vaxxers’ activity on Facebook and Twitter is similar, focusing mainly on distrust of the government and allegations regarding vaccination safety and effectiveness. Anti-vaxxers on TikTok mainly focus on personal freedom. |
| Yousefinaghani et al | 1/7,2020-1/3,2021 | / | Twitter |  | The main topics in positive tweets included hope, support, and faith, while negative tweets were usually related to fear, discouragement, anger, and politics. News organizations were found to be the most active content writers in positive content. |
| Islam et al | 12/31,2019-11/30,2020 | 52 countries | Google, Facebook, YouTube, Twitter, fact-check agencies | Manual coding | Six hundred thirty-seven items were identified as rumors and conspiracy theories related to the COVID-19 vaccine from 52 countries, of which 91% were rumors, and 9% were conspiracy theories. Of 578 rumors, 36% were related to vaccine development, availability, and access, 20% were related to morbidity and mortality, 8% to safety, efficacy, and acceptance, and the rest were other categories. Of the 637 items, 5% were true, 83% were false, 10% were misleading, and 2% were exaggerated. |
| Thelwall | 12/5,2020-3/21,2021 | US, UK, Canada, India, Australia, South Africa, Ireland, Nigeria | Twitter | Manual coding; Word Association Thematic Analysis (WATA) | The differing relevance of independent medical experts is a potential concern, given that coherent public health messages are necessary for vaccination programs. |
| Scannell et al | 7/14-7/23, 2020 | US | Twitter | Manual coding | Anti-Vaccine messages predominantly used Anecdotal stories, Humor/Sarcasm, and Celebrity figures as persuasion techniques, while Pro-Vaccine messages primarily used Information, Celebrity figures, and Participation. |
| ***Other algorithms (n=12)*** | | | | | |
| Jarynowski et al | 12/9,2020-4/17,2021 | Russia | Telegram group | Bidirectional Encoder Representations from Transformers (BERT) | Russian Telegram users reported mostly pain, fever, and fatigue. |
| Breeze et al | 2/28-7/23,2020 | US and UK | Mail Online | A corpus-assisted discourse analysis approach | The appeal to expert knowledge is no longer a foolproof means of guaranteeing that information is important in the public arena. |
| Boucher et al | 11/19-11/26,2020 | / | Twitter | Social network analysis (SNA) and unsupervised machine learning (DistilBERT) | The main themes driving the vaccine hesitancy conversation were concerns about safety, efficacy, freedom, and mistrust in institutions. Mistrust in institutions has emerged as a predominant theme of vaccine hesitancy conversations on Twitter. |
| Monselise et al | 12/16,2020-2/13,2021 | US | Twitter | Non-negative matrix factorization (NMF) | The most important topics with the highest topic ratio were the vaccination of frontline workers, access to vaccines by signing up online, and the South African variant. |
| Mourad et al | 2/1-4/30,2020 | Arab region | Twitter | Bidirectional Encoder Representations from Transformers (BERT) | The most prominent topic tweeted for the English dataset was COVID-19 statistics, and the last was a conspiracy theory. Governmental measures were the most tweeted topic for both French and Arabic datasets. |
| Teng et al | / | / | YouTube | SAS Text Miner | Polarized views on vaccines existed in the social media ecology of public discourse, with a majority of people unwilling to get vaccinated against COVID-19. Reasons behind vaccine hesitancy included concerns about vaccine safety, potential side effects, and lack of trust in government and pharmaceutical companies. |
| Baj-Rogowska et al | 5/1-5/30,2021 | Global | Twitter | An algorithm implemented in the WordStat software | COVID-19 vaccine uptake mostly depends on the dimensions defined as Awareness and Access to the vaccine. Awareness covers the availability of a wide range of actual and detailed information regarding vaccines in the population, such as immunization schedules, vaccine side effects, safety, and efficacy. |
| Chandrasekaran et al | 1/1,2020-4/30,2021 | / | Twitter | CorEx | The most tweeted topic about COVID-19 vaccination was related to vaccination policy, specifically whether vaccines needed to be mandated or optional, followed by vaccine hesitancy and post-vaccination symptoms and effects. |
| Hwang et al | 3-5,2021 | / | Twitter | Structural Topic Modeling (STM) | The most prominent topic that emerged on Twitter regarding a COVID-19 vaccine was "other infectious diseases," followed by "vaccine safety concerns" and "conspiracy theory." The positive discourse was more likely to interact with verified sources such as scientists/medical sources and the media/journalists. In contrast, negative discourse tended to interact with politicians and online influencers. |
| Kumar et al | 1/1-12/14,2021 | US | Reddit | Structural Topic Modeling (STM) | There is an association between positive vaccine developments and an increase in the discussion of COVID-19 vaccine misinformation and a relationship between development setbacks and reduced misinformation discussion. |
| Lanyi et al | 11/30,2020-8/15,2021 | UK | Twitter | AI | Safety concerns, mistrust of government and pharmaceutical companies, and accessibility issues as key barriers limiting vaccine uptake. |
| Lian et al | 12/1,2020-8/1,2021 | US | Twitter | Named Entity Recognition (NER) | Sore to the touch, fatigue, and headache are the three most common adverse effects of all three COVID-19 vaccines in the US. |
| **Behavioral analysis (n=3)** | | | | | |
| Merrick et al | 6-7,2020 | US | Google Trends | GL analysis | Search interest related to vaccine misinformation, general information, and access-seeking changed in relation to events occurring throughout the pandemic. |
| Blane et al | 12,2020-1,2021 | / | Twitter | Organization Risk Analyzer - PRO Software | Pro-vaccine users primarily used positive maneuvers such as exciting and explaining messages to encourage vaccination and backed leaders within their group. Anti-vaccine users relied on negative maneuvers to dismay and distorted messages with narratives on side effects and death. They attempted to neutralize the effectiveness of the leaders within the pro-vaccine community. |
| Liu et al | 11/1-11/22,2020 | / | Twitter | Manual coding based on the capability, opportunity, motivation - behavior model | The positive behavioral intention was affected by reduced risk of infection, socioeconomic recovery, and return to normal life. In contrast, the negative behavioral intention was associated with misconceptions about vaccines and diseases, trust in natural immunity, distrust in government and vaccines, and lack of knowledge. |
| **Dissemination and engagement analysis (n=9)** | | | | | |
| Mir et al | 1/12-2/13,2021 | / | Twitter | / | The tweets that express positive sentiment have the highest impact compared to the “negative” and “neutral” ones. |
| Olszowski et al | 7/26-12/11,2021 | Poland | Twitter | Software NodeXL | Vaccination mandate opponents are vocal and more mobilized to participate, either as original authors or information diffusers. Vaccination mandate advocates are consistently more numerous but less engaged and less mobilized to “preach” their stances. |
| Hagen et al | 7/1-7/31,2020 | / | Twitter | PageRank algorithm | The most influential Twitter actors were not scientists and medical experts but partisan actors and anti-vaxxers. The antivaccine sentiment was especially salient in the political right cluster. |
| Hwang et al | 3-5,2021 | / | Twitter | Network Analysis | Positive vaccine discourse tended to be circulated by the network of scientists/medical sources and media/journalists. Other networks, including political sources, online influencers, and suspended accounts, spread negative vaccine sentiment. |
| Xie et al | 3/5,2020-1/25,2021 | US | Twitter | DeepFace algorithm | Twitter users from the east, west, and southern states of the US, as well as male users and users in the age group 30-49 years, were more likely to discuss COVID-19 vaccines on Twitter. |
| Pascual-Ferrá et al | 12/29,2019-1/2,2021 | US | Google，YouTube， Facebook, Instagram, Reddit | Machine learning (Communalytic) | Most vaccine-related posts on Facebook, Instagram, and Reddit had low toxicity scores. No evidence of negative dominance of vaccine-related content in this study. |
| Kalichman et al | 2/1-5/31,2020 | US | Facebook | Numeric values on the extent of dissemination | COVID-19 posts were more widely disseminated and showed greater influence than non-COVID-19 posts. Early COVID-19 posts concerned mistrust of vaccine safety and conspiracy theories. |
| Herrera-Peco et al | 12/14-12/28,2020 | Spanish | Twitter | Social network analysis (betweenness centrality score) | The most influential users were mainly accounts of official organizations. Healthcare professionals' four highest active professional groups were pharmacists, nurses, physicians, and psychologists. |
| Islam et al | 12/31,2019-11/30,2020 | 52 countries | Google, Google Fact Check, Facebook, YouTube, Twitter, websites | / | Facebook was the most prevalent social media, followed by Twitter. Three waves of rumors and conspiracy theories were consistent with a Google search on the COVID-19 vaccine. |
| **Information quality analysis (n=7)** | | | | | |
| Savolainen | 1/1-2/7,2021 | / | Reddit | Manually coding | In assessing the credibility of mis/disinformation, the authors’ qualities such as poor reputation/incompetency/dishonesty are particularly significant. The credibility of anti-vaxxers as creators of vaccine-related information is very low in the eyes of pro-vax online discussion contributors. |
| Li et al | 7/21,2021 | / | YouTube | The modified DISCERN score, the modified JAMA score, and the COVID-19 Vaccine Score | Approximately 11% of YouTube s most viewed videos on COVID-19 vaccines contradicted the reference standard. Videos containing non-factual information had significantly lower mDISCERN, mJAMA, and CVS scores compared with videos with factual information. |
| Kocyigit et al | / | / | YouTube | Manually coding | Two-thirds of the videos were of high quality and relatively few low-quality videos were presented. The sources of high-quality videos were pharmaceutical company, pharmacist, society-organization, and academic. While news provided a high percentage of low-quality videos. |
| Lentzen et al | / | / | Twitter and Instagram | Manually coding | Only 3 to 7% were rated by excellent educational and validatable content. |
| Yeung et al | 7/8-9/29,2021 | 56 countries | Website | Manually coding | COVID-19 vaccination FAQs websites provide good quality information, but more effort should be paid to make them more readable and updated. |
| Zhao et al | 7/1,2020 | China | iQiYi | Manually coding | Most videos received high scores on Global Quality Scale (GQS), and all the videos partly adhere to Health on the Net Foundation Code of Conduct (HONCode) and DISCERN principles. |
| Herrera-Peco et al | 12/14-12/28,2020 | Spanish | Twitter | Manually coding | Only 5% of tweets provided external links that allowed independent confirmation. 71% of tweets had problems with inappropriate writing and spelling or grammar, resulting in the impression of low reliability. |
