# Supplementary material for: Applications of Social Media and Digital Technologies in COVID-19 Vaccination: Scoping Review
Source: J Med Internet Res. 2023 Feb 10;25:e40057. doi: 10.2196/40057 (PMC9924059; doi:10.2196/40057)
Supplement: Multimedia Appendix 5 [file jmir_v25i1e40057_app5.docx]

**Multimedia Appendix 5.** Characteristics, applications, innovation features, and generalizability of the included digital technology articles (n=64).

| **Reference** | **Data source** | **Digital technology** | | **Details of the applications** | **Innovation feature** ^a^ | **Generalizability** ^b^ |
| --- | --- | --- | --- | --- | --- | --- |
| ***Strategy of COVID-19 vaccination (n=9)*** | | | | | | |
| Romeo L et al | Italian Federation of General Practitioners dataset (Electronic Health Record data) | Algorithm | Unified Hierarchical XGBoost model | A machine learning algorithm, namely Hierarchical Priority Classification eXtreme Gradient Boosting, is proposed for priority classification for COVID-19 vaccine administration using the Italian Federation of General Practitioners dataset that contains Electronic Health Record data of 17k patients. The machine learning methodology comprises two hierarchical layers, layer A for predicting vulnerable class and layer B for age-dependent class. | Complement | Local (data source issues) |
| Sulis E et al | Not mention | Algorithm | Genetic Algorithms | The genetic algorithm was adopted to find the optimal parameters of the vaccination campaign and vaccination sequences by people groups based on S.I.s.a.R. model to reduce the number of symptomatic infected people. | Complement | Global |
| Lende M et al | COVID-19 data from Our World in Data; Provisional COVID-19 death counts and contributors | Algorithm | Fuzzy logic system | Use datasets to identify if there exists a relationship or dependence among COVID-19 infections and demographic features like age, sex or other underlying co-morbidities, determine parameters for the basis of prioritization and form synthetic data. Rank regions through fuzzy logic system. | Supportive | Global |
| Couto RC et al | 49197 patients from 336 Brazilian hospitals | Algorithm | Random Forest, XGBoost, and Logistic Regression | Machine learning algorithms were used to develop a predictive model for in-hospital mortality in patients with COVID-19 infection. 70% of the data set was used as training data, and the remaining 30% was used as test data. | Supportive | Global |
| Talan R | Data on percentage of vaccinated people per country from WHO; Worldometer coronavirus daily dataset; coronavirus summary dataset | Algorithm | Long Short Term Memory (LSTM) and Auto-Regressive Integrated Moving Average Model (ARIMA) | Construct a model utilizing Auto-Regressive Integrated Moving Average Model (ARIMA) and Long Short Term Memory (LSTM) to forecast the trend of COVID-19 prevalence in the different states of India, Delhi, Kerala, Maharashtra and India as a whole. | Complement | Global |
| Trad F et al | Virtual generated data on COVID-19 vaccination | Algorithm | Reinforcement Learning | Reinforcement Learning (RL) was used to predict the evolution of COVID-19 inside a country with virus parameters, different control measures, and different vaccine distribution strategies to find vaccine distribution strategies. | Complement | Global |
| Hughes et al | Not mention | Algorithm | Genetic programming system | Genetic programming system is used to simulate and test mitigation strategies for an epidemic on a given social network graph. Strategy evaluation and selection of the best strategy through the SEIR epidemic model. | Complement | Global |
| Jadidi et al | Contact tracing data over cellular networks and Bluetooth signals | Algorithm | Disease propagation graph | A disease propagation graph is used to model the transmission of the disease between individuals for targeted vaccination, where nodes represent individuals. Vaccinate the node with the highest susceptibility after calculating the metrics for all nodes, then remove the selected node and update the graph until all available vaccines are administered. | Innovative | Local (data source issues) |
| Rocha et al | National Registry of Health Facilities, OpenStreetMap, WorldPop, Mobile phone base stations, SIVEP-Gripe, IBGE population projections | Algorithm | Spatial artificial intelligence | Spatial artificial intelligence is used to identify the geographic location of Brazil’s primary care centers, and estimate the population registered with the vaccination rooms and the straight-line distance between PCCs and cell towers connected to a mobile data network. Identify priority regions in accordance through combining information on populations within the COVID-19 vaccination plan’s target age group and their location, COVID-19 situation, and internet access in vaccination rooms | Innovative | Local (data source issues) |
| ***Distribution and delivery of COVID-19 vaccines (n=22)*** | | | | | | |
| Rathee G et al | Not applicable | Blockchain | No specifics | The level-wise blockchain network is used to ensure security among IoT devices while distributing the vaccines. | Supportive | Local (technical issues) |
| Musamih A et al | Not applicable | Blockchain | Ethereum | Ethereum blockchain is a solution for managing COVID-19 vaccine distribution and delivery data. Smart contracts to automate the traceability of COVID-19 vaccines while ensuring data provenance, transparency, security, and accountability. | Supportive | Local (technical issues) |
| Das AK et al | Not applicable | Algorithm; Blockchain; Internet of things (IoT) | No specifics | Various transactions related to vaccine requests, orders, distribution, and tracking are put into the blockchain in the form of blocks. The information stored into the blocks in the blockchain is analyzed using AI-based big data. | Supportive | Local (technical issues) |
| Shukla S et al | Details on partner long-term care facilities (LTCFs) by state, CVS depot locations capable of providing COVID-19 vaccine, distances between depot-LTCF pairs, and distance threshold required by the CDC; Capacity constraints provided by the vaccine provider; Vaccine provider partnerships | Algorithm | Spatial-geographical model; Minimum-cost flow problem; Self-designed scheduling algorithm | A subset of the CVS depots is uniformly selected through a spatial-geographical model to form a logistical distribution hub. Propose an optimal solution to the depot-LTCF (long-term care facility) assignment problem by modeling the problem as a minimum-cost flow problem. Given the optimal assignments, they devise a scheduling algorithm to minimize days needed to vaccinate LTCFs in every state and compute schedules for the clinics to orchestrate the two doses at the recommended dosing interval. The algorithm further optimized scheduling by strategically clustering several small LTCFs together and pairing them with a large LTCF to determine which group of LTCFs could be vaccinated on the same day, given capacity, distance, and supply constraints. | Complement | Local (healthcare infrastructure issues) |
| Verma A et al | Not applicable | Blockchain; unmanned aerial vehicles | Smart contracts; 5G-tactile internet (5G-TI) enabled UAV communication networks | The scheme supports resilient vaccine distributions through 5G-TI-based unmanned aerial vehicle communication networks (UAVCN) in a phased manner at government-designated nodal centers (NCs). Once vaccines are made available at the NC warehouse, the blockchain ensures timestamped documentation of vaccinated persons with chronology, auditability, and transparency of supply-chain checkpoints from VPW to the NC. Through smart contracts (SCs), priority groups can be formed for vaccination based on age, healthcare workers, and general commodities. | Innovative | Local (technical issues) |
| Wei X et al | Time series data on the number of confirmed cases in five US states | Algorithm | DDQN, A2C, TRPO, ACKTR, and LinUCB model | An End-to-End vaccine distribution model is proposed by combining the Deep Reinforcement Learning model and LinUCB algorithm to get an optimistic allocation strategy. | Supportive | Global |
| Akshita V et al | Not applicable | Blockchain | No specifics | A blockchain-based system was implemented to trace the registration, transparency, storage, and delivery of COVID-19 vaccine. In healthcare official module, using blockchain makes the system tamper-proof and maintains a proper record of all the transactions which has taken place. The admin module stores each transaction's hash value, making it easy to track all the operations in the app, like the test details, user registration, health care official registration, vaccine booking, and count of vaccines available in the hospital. | Supportive | Local (technical issues) |
| Antal C et al | Not applicable | Blockchain | Ethereum; Smart contract | Blockchain technology is used to guarantee data integrity and immutability of beneficiary registration for vaccination, avoiding identity thefts and impersonations. Smart contracts are defined to monitor and track the proper vaccine distribution conditions against the safe handling rules defined by vaccine producers. | Supportive | Local (technical issues) |
| Barajas M et al | Number of people greater than 18 years, priority groups, vaccines ordered by jurisdiction, vaccine shipping, administration of vaccines, tracking vaccine distribution | Algorithm | Long Short-Term Memory (LSTM) | A vaccine distribution demand forecasting system based on the long short-term memory neural network was proposed to determine where and how the vaccine should be distributed. | Complement | Global |
| Chauhan H et al | Hypothetical scenario | Blockchain | Smart contract | The distributed ledger-based framework was used for immutable data, transparency, and efficiency of the registration process for COVID-19 vaccination to avoid counterfeit and identification theft. Smart contract-enabled framework was used for self-administering the vaccine distribution constraints in the cold chain regarding the fulfillment of COVID-19 vaccine. The framework for vaccine supply chain management will enable the features of tamper-proof, person identification, and avoidance of counterfeiting. Based on these proposed frameworks, the complete process of the patients' vaccination from registration to successfully being vaccinated, as well as any post-vaccination symptoms or side effects, will be monitored and stored as immutable data disabling any tampering by third parties. | Supportive | Local (technical issues) |
| Chung HM et al | Hypothetical scenario | Blockchain | Smart contract | Smart contrast was used to improve the supply chain of COVID-19 vaccines, such as prevention of errors, protection from fraud and counterfeit products, identifying actors and locations on the timeline, and managing flows and processes, among others. | Supportive | Local (technical issues) |
| Enriko | Not mention | Internet of things (IoT) | No specifics | The Internet of Things (IoT) technology was used to enhance Indonesia's existing cold chain vaccine distribution system. With this technology, vaccine distribution, like location and temperature, can be tracked precisely by deploying a LoRa-based tracker and monitoring sensor in the distribution trucks, and vaccine conditions can be maintained by alerting the responsible personnel. | Supportive | Global |
| Goodarzian F et al | COVID-19 vaccination related data of Tehran/Iran | Algorithm | Gray wolf optimization, Variable neighborhood search | Three meta-heuristics - Gray wolf optimization (GWO), Variable neighborhood search (VNS), and Modified Gray wolf optimization (MGWO) - were used to solve different problems of COVID-19 vaccines, including allocation, location, routing, distribution, supply chain network, inventory management, production, etc. | Innovative | Global |
| Jain R et al | Not applicable | Algorithm; Internet of things (IoT) | No specifics | ML regression was used to predict the temperature of the next 20 minutes by analyzing a series of real-time data of the temperature in COVID-19 vaccine storage, which would be obtained by a temperature sensor and stored in the cloud. | Complement | Global |
| Kamran MA et al | COVID-19 vaccination related data of Tehran/Iran | Algorithm | Whale Optimization Algorithm, Variable Neighborhood Search | Whale Optimization Algorithm (WOA), Variable Neighborhood Search (VNS), and Modified Whale Optimization Algorithm (MVWOA) algorithms were used to minimize vaccine supply chain costs, maximize student desirability for vaccination, and maximize justice in vaccine distribution. | Innovative | Global |
| Kaur N et al | COVID-19 data of 31 COVAX countries from Our World in Data | Algorithm | Artificial Neural Network (ANN); Convolutional Neural Network (CNN); Linear Regression | This predictive model is leveraged to design the optimal vaccine distribution strategy that simultaneously minimizes the resulting risks while maximizing the vaccination coverage in these countries targeted by COVAX. | Complement | Global |
| Markhorst B et al | COVID-19 vaccination related data of Dutch | Algorithm | Heuristic algorithm | A priority list of COVID-19 vaccination groups different inhabitants into different vaccines and vaccination moments. A Decision Support System supported by a heuristic algorithm was proposed to optimize the performance measure of per-class waiting time by determining the optimal placement of medical hubs and the division of vaccines and healthcare personnel. | Complement | Local (data source issues) |
| Meghla TI et al | Not applicable | Algorithm; Blockchain | Blockchain, Machine Learning (ML) Regression, ML Classification | Blockchain is used to ensure that information on vaccine supply is stored and processed correctly. Machine Learning was used to forecast vaccination demand and the most appropriate vaccine for a particular geographical area. Two different types of machine learning techniques have been used: ML Regression for demand forecasting, and ML Classification to select appropriate vaccines. | Complement | Local (technical issues) |
| Rahman A et al | Not applicable | Blockchain; IoT | SDN-IoT model based on blockchain | The SDN-IoT model can help to determine the priority of vaccination by population groups and risks of virus transmission and to improve the supply of vaccines, vaccination information records, vaccination eligibility decisions, storage, and distribution of vaccines. | Complement | Global |
| Rotbi MF et al | Not applicable | Blockchain | Smart contract | Smart contract (SC) could help to ensure that desirable conditions are maintained during the storage and transportation of COVID-19 vaccines throughout the cold chain, as well as issue warning signs if sensors detect any abnormalities in the handling of vaccines. | Supportive | Local (technical issues) |
| Verma A et al | Not applicable | Blockchain; unmanned aerial vehicles | Smart contracts; 6G enhanced ultra-reliable low latency communication | Blockchain technology was used to assist unmanned aerial vehicles (UAVs) in COVID-19 vaccine distribution by assuring transparency in the vaccine supply chain from vaccine manufacturing labs to vaccination centers. Blockchain also mitigated security attacks on UAV swarms. | Complement | Local (technical issues) |
| Davahli et a | Dataset from the US Centers for Disease and Prevention website | Algorithm | Unsupervised self-organizing map; Long Short-Term Memory (LSTM) model; Recurrent neural network (RNN) model；Stochastic Mixture Density Network (MDN) model | An unsupervised self-organizing map is used to categorize all US states based on the similarity of their effective reproduction numbers. Then train a deterministic Long/Short-Term Memory (LSTM) model, recurrent neural network (RNN) model, and stochastic Mixture Density Network (MDN) model with the data from the earliest outbreak states in each group. | Complement | Global |
| ***Model prediction of COVID-19 vaccination (n=6)*** | | | | | | |
| Sujatha R et al | Vaccine adverse event reporting system (VAERS) | Algorithm | Logistic Regression, Adaboost, Decision Tree (DT), and Random Forest (RF) | Machine learning techniques and algorithms were used to analyze data from vaccine adverse event reporting system (VAERS) and to predict the working of COVID-19 vaccines on specific age groups developed by significant vaccine manufacturers. | Supportive | Global |
| Bashir S et al | Publicly available data of COVID-19 vaccines | Algorithm | Long Short Term Memory (LSTM) | An LSTM-based model is trained to predict COVID-19 vaccination coverage in Pakistan. | Complement | Global |
| Hua F et al | A dataset contains various COVID-19 World Vaccination Process information | Algorithm | Long Short Term Memory (LSTM), CNN-LSTM, VAE-LSTM, DeepAR | These four algorithms (LSTM, CNN-LSTM, VAE-LSTM, and DeepAR) were used to predict world vaccination rates. | Supportive | Global |
| Ji Z et al | COVID-19 World Vaccination Progress from Our World in Data; COVID global data from WHO | Algorithm | Linear regression, Decision Tree (DT), Support Vector Machines (SVM) | Linear regression was used to forecast vaccination process and determine cause-and-effect relationships between variables. Decision Trees (DT) and Support Vector Machines (SVM) were used to predict the future number of vaccines based on the database. | Supportive | Global |
| Zhou X et al | Daily first-dose vaccination in the United States from CDC | Algorithm | Auto-regressive model, linear regression, boost and random forest | Auto-regressive model, and Machine Learning such as boost classification and random-forest algorithm were used to forecast the future COVID-19 vaccine uptake rate. | Complement | Local (data source issues) |
| Bandyopadhyay et al | Kaggle repository | Algorithm | Random Forest, Extra Trees, Gradient Boosting, AdaBoost, and XGB | Ensemble-based machine learning Regressor models such as Random Forest, Extra Trees, Gradient Boosting, AdaBoost, and XGB, were used to predict daily COVID-19 vaccination progress. | Innovative | Global |
| ***COVID-19 vaccination services (n=11)*** | | | | | | |
| Asgary A et al | A dataset from a drive-through mass vaccination simulation tool | Algorithm | AnyLogic model and a trained neural network | AnyLogic model, a hybrid model consisting of a discrete event and an agent-based simulation to simulate the service process of vaccination for each lane from registration to recovery. A trained neural network to predict the outputs of the simulation (the number of people to be vaccinated and the average time it takes for vaccination). | Complement | Global |
| Alismail S et al | 54 official COVID-19 vaccine registration and  management websites by the CDC | Online tool | WAVE, A Checker, SortSite11 evaluation tools | Employ Web accessibility evaluation tools to examine the accessibility of 54 official state and territory COVID-19 vaccine registration websites in the US and ensure that users with disabilities can independently schedule vaccination appointments. | Supportive | Global |
| Noack EM et al | Summaries of product characteristics published by vaccine producers; vaccine safety information; Process flows at German vaccination centers | mHealth | Self-developed multilingual app | This multilingual app in 40 languages facilitates communications between healthcare professionals and vaccination candidates with limited local language skills, including providing effectively legally compliant information about the risks and benefits of the COVID-19 vaccination, obtaining informed consent, and guiding vaccination candidates through the vaccination process. | Supportive | Global |
| Batavia P et al | The VGG Face Dataset, the FaceScrub Dataset, and the “Labeled Faces in the Wild” dataset | mHealth | APP | This App 1) assists users in navigating through the waiting, vaccination, and monitoring room using the indoor navigation map of the vaccination center; 2) helps maintain the social distancing norms by highlighting the crowded place and corroborates the compulsory use of masks by face recognition; 3) applies an in-application barcode scanner to capture the vaccine and vaccination-related info. | Complement | Local (technical issues) |
| Cabezas X et al | Not applicable | Algorithm | Accelerated Dual Ascent (ADA) | This algorithm stores patients’ records in a central data warehouse using sensors, scheduling the patients to the more suitable vaccination centers that meet the immediate needs of patients. | Complement | Local (technical issues) |
| Demirel DY et al | Not applicable | mHealth | APP (ProvVa) | ProvVacT mobile application is developed for vaccine tracking, which enables users to track their vaccination dates and reminds users before their next vaccination date. | Supportive | Global |
| Ford KL et al | Not applicable | mHealth | Personalized message/e-mail | AI reinforcement learning platform was used to select, assemble, and deliver the right COVID-19 vaccination promotion message components for individuals through message/e-mail based on their characteristics and behaviors. This method enables personalized digital health communication to increase COVID-19 vaccination in underserved populations. | Supportive | Global |
| Fuentes AF et al | An electronic Health records data warehouse in Santiago de Cali | Cloud storage | BigQuery;  Geocoding system;  BackEnd Technologies | BigQuery was used to interact with the HER data warehouse to obtain patients' electronic health records and health status and identify and characterize patients who meet the prioritization criteria for Covid-19 vaccination. The Geocoding system was used to execute geocoding processes of identified patients. BackEnd Technologies were integrated to develop the BackEnd Layer of the Covid-19 Vaccination Tracking and Control platform. | Complement | Local (data source issues) |
| Jemmali M et al | Not mention | Algorithm | A series of intelligent algorithms | A series of intelligent algorithms - Decreasing order-based algorithm (DA), Iterative random algorithm (IR), Clustering algorithm based on two or three sets, et al - are used to help design a smart parking system. The smart parking system will help the health crisis management committee vaccinate the largest number of people within the minimum period while ensuring that all precautionary measures are followed through a set of algorithms. These algorithms seek to ensure a uniform distribution of persons in parking. | Innovative | Global |
| Kumar A | Address information of unvaccinated patients in Manhattan | Algorithm | K-means clustering | K-means clustering machine learning algorithm was used to process the addresses of patients unvaccinated against COVID-19, group them into clusters, and export these clusters into a standard database format and onto a map to optimize the door-to-door COVID-19 vaccination route of the visiting doctors, maximizing efficiency and minimizing vaccine waste | Complement | Global |
| Pilati F et al | Not applicable | Digital twin; mHealth | Static simulation model and digital technology (APP) | The APP will measure, record, and send the starting time and duration of each process of the APP user's ongoing COVID-19 vaccination to the managers. These time data will be input into a Simulation Model to perform tests to identify the best strategy to implement the COVID-19 vaccination campaign with fewer resources and less waiting time. | Complement | Local (ethical and technical issues) |
| ***Certification of COVID-19 vaccination (n=13)*** | | | | | | |
| Abubakar M et al | Not applicable | Blockchain | Ethereum; Smart contract | Ethereum blockchain system and smart contract technology were used to build a secure and scalable decentralized architecture for COVID-19 vaccination certificate creation and verification. Verifiable Credentials were utilized to encrypt and decrypt sensitive information, and smart contracts were used to control access and to provide on-chain verification and validation of the user and issuers Decentralized Identifiers. InterPlanetary File System was leveraged to decentralize the storage of vaccination certificates and medical tests. | Complement | Local (transactions cost and key management issues) |
| Ajakwe S et al | Not applicable | Algorithm; Biometrics | Facial recognition; Mask wearing detection technology using Yolov5 deep learning model | An IT-convergence solution that incorporates facial recognition and mask-wearing detection technology was used to distinguish between vaccinated and non-vaccinated individuals in real time and initiate strict and appropriate compliance directives and consequent denial of access to certain places. | Innovative | Local (ethical and privacy issues) |
| Arif M et al | 5000 vaccine certificates over different countries | Algorithm | Dense Convolutional Networks (DenseNets) | Detect fake vaccine certificates through a bot powered by Artificial Intelligence and neurologically powered by Deep Learning | Innovative | Local (technical issues) |
| Jorge H | Hypothetical scenario | Blockchain | Smart contract | Smart contrast was used to deal with some identified healthcare challenges and issues, including privacy, unique identity, and scalability. A blockchain global network was proposed to support smart contracts for scalability, privacy, and security, reducing latency and providing fast and real-time access to COVID-19 vaccination process, avoiding a single point of failure. | Complement | Global |
| Faroug A L et al | Not applicable | Blockchain | Ethereum | Hyperledger Fabric was used to create patients' electronic health tables. A system based on the Ethereum platform was developed to track and efficiently verify the vaccination records for people who want to travel abroad during the ongoing COVID-19 pandemic. | Supportive | Global |
| Kanimozhi S et al | Not applicable | Algorithm | Convolutional Neural Networks (CNNs) | Deep Learning in the form of Convolutional Neural Networks (CNNs) was used to carry out the face recognition process, which was matched to individuals' COVID-19 vaccination status. | Complement | Local (ethical and privacy issues) |
| Khan D et al | Not applicable | Blockchain | Hash Algorithm | A novel blockchain-based system was used to provide a verifiable digital COVID-19 Immunity Certificate. This certificate can address the issues in trackability, and traceability of the data embedded in the COVID-19 immunity certificate to eradicate COVID-19 vaccination immunity certificate fraud and to establish trust in the COVID-19 certificate bearer. | Innovative | Global |
| Lee HA | Not applicable | Blockchain | Smart contract | The blockchain decentralized mechanism was used to build an open and anticounterfeiting information platform for vaccination passports. The SC was used for authorization and authentication to achieve hierarchical management of various international hospitals and people receiving injections. PoA was used to connect government agencies of various countries while countries jointly maintained the verification of blocks. | Innovative | Global |
| Loss S et al | Not applicable | Blockchain | A blockchain adaptor (Canis Major) | Canis Major and FIWARE technology were used to allow vaccination control system of any country to submit vaccination certificates as transactions in NGSI format from the FIWARE middleware components to any Distributed Ledger Technology (DLT) implementation supported by the proposed Canis Major Generic Enabler (GE) without any intervention of humans. | Innovative | Global |
| Lupu C et al | Not applicable | Biometrics | Iris recognition | Iris recognition was used to identify whether the user had been vaccinated against COVID-19, recovered from COVID-19, or received an RT-PCR test to determine the presence or absence of SARS-CoV-2 virus. | Innovative | Local (ethical and privacy issues) |
| Mahamud S et al | Not applicable | Blockchain | Smart contract | The blockchain technology goes through the vaccination system after user registration, identifying and monitoring the COVID-19 vaccination process of every user by specific registration ID, then generates a COVID-19 vaccination certification and sends it to fully vaccinated users. | Complement | Global |
| Eisenstadt et al | Not applicable | Blockchain, mHealth | A mobile phone app; Ethereum | A mobile phone app and requisite decentralized server architecture that facilitates instant verification of tamper-proof test results. The architecture relies upon the 2019 World Wide Web Consortium standard “Verifiable Credentials”, Tim Berners-Lee’s decentralized personal data platform “Solid”, and a Consortium Ethereum-based blockchain. | Innovative | Local (biological, ethical, and technical issues) |
| Wilson S | Not applicable | mHealth | A digital Yellow Card on mobile phones | A digital Yellow Card on mobile phones securely could hold verified records of vaccination and other attributes that a person accumulates as they make their way through the world. The design has practical benefits for low-doc persons' digital engagement and privacy. | Innovative | Global |
| ***Post-vaccination surveillance (n=3)*** | | | | | | |
| Yamazaki S et al | Adverse events after vaccination self-reported by health care workers at Chiba University Hospital | mHealth | Mobile app (Respon:sum (Smart119 Inc., Chiba, JP)) | Healthcare workers (HCWs) vaccinated with Comirnaty® were asked to report adverse events within 14 days after vaccination through the application software respon: sum according to severity. | Complement | Global |
| Di Filippo M et al | Academic subjects vaccinated at the Milano-Bicocca University hub for COVID-19 | mHealth | Mobile app (vaxEffect@UniMiB) | The mobile app asks users to send their medical history and vaccination data, as well as adverse reactions that occurred within seven days of vaccination so that the dynamics of adverse drug reactions (ADRs) can be tracked for each respondent. The application sends the data via the Web to the application server. After receiving all user data, the server saves it in a SQL database server and sends push notifications to the mobile application via Firebase Cloud Messaging (FCM) to remind patients to submit their vaccine and ADR data. | Complement | Local (technical issues) |
| Wang T et al | Not applicable | Blockchain | Smart contract | The health code blockchain was used to track and monitor the holder’s health and vaccination status, action trajectory, and protection status. Infection status and severity if infected were recorded in blockchain and compared by different COVID-19 vaccination statuses to maintain the vaccine effectiveness. | Innovative | Local (technical issues) |

Note: ^a^ Innovation features of digital technologies/solutions are classified into four groups ^6^: 1) supportive - support the performance of existing technologies; 2) complement - strengthen the performance of existing technologies; 3) innovative - offer new possibilities unavailable before; 4) substitutive - replace old technologies.

^b^ Generalizability of digital technologies/solutions ^7^ covers three groups: 1) not possible - strict bond to the context in which they are developed; 2) local - its scalability limited to a local regional context for normative, legislative, ethical, or technical reasons; 3) global - no barriers to scalability for global adoption.
